# Supplementary figures and images for: C. elegans CLASP/CLS-2 negatively regulates membrane ingression throughout the oocyte cortex and is required for polar body extrusion
Source: PLoS Genet. 2020 Oct 7;16(10):e1008751. doi: 10.1371/journal.pgen.1008751 (PMC7571700; doi:10.1371/journal.pgen.1008751)

S1 Fig

# A

CLS-2::GFP ; mCherry::H2B

— 10μm

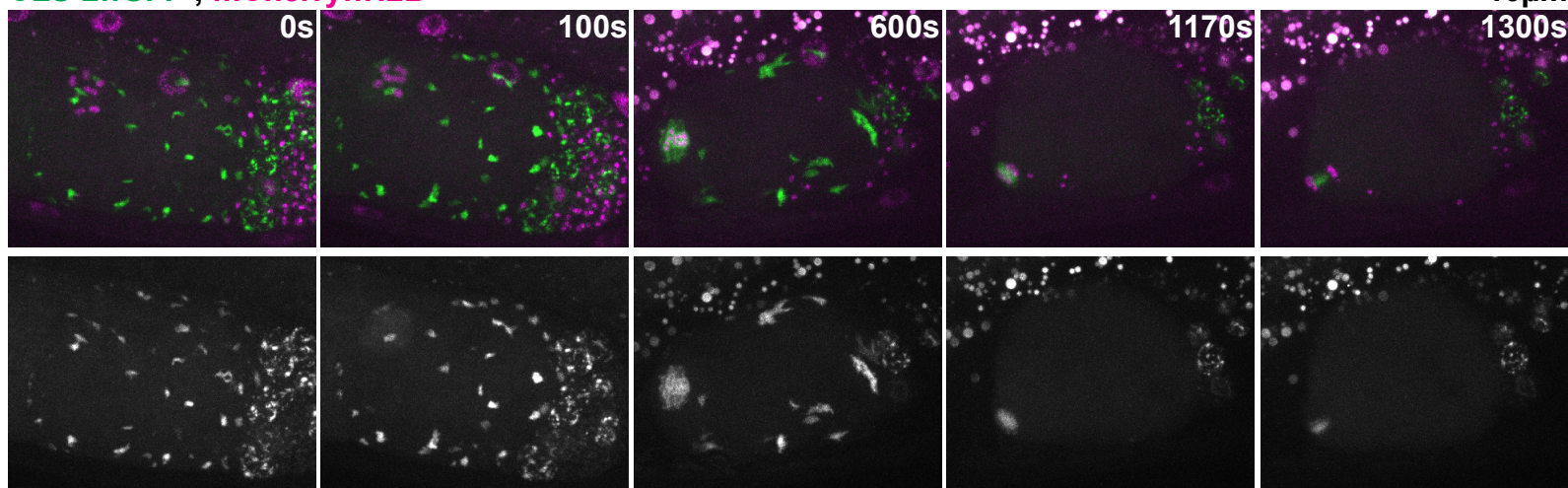

# B

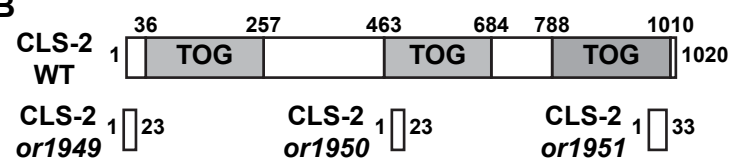

# C

GFP::Tubulin ; mCherry::H2B

— 5μm

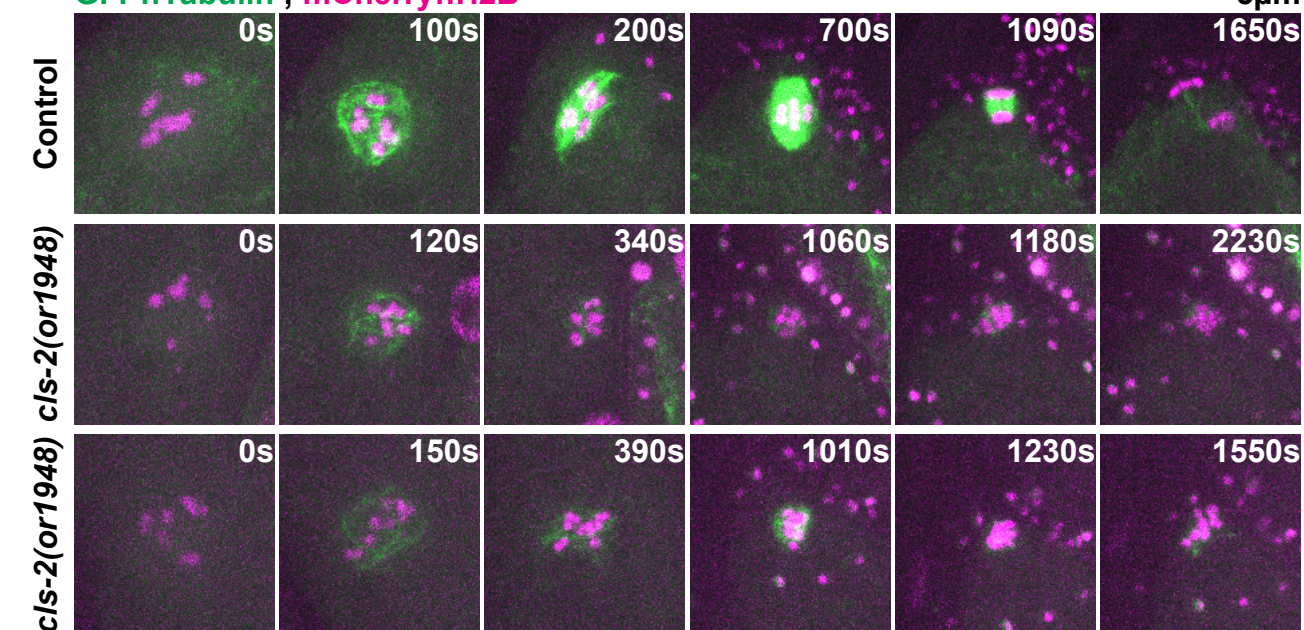

Supplement: S1 Fig — (A) In utero time-lapse spinning disk confocal images of CLS-2::GFP and mCherry::H2B. (B) Protein domain maps of wild type CLS-2 and CRISPR-generated cls-2 alleles or1949, or1950, and or1951. Each mutation results in multiple early stop codons before the first TOG domain, with the first stop codon indicated. (C) In utero time-lapse spinning disk confocal images of control and cls-2 mutant oocytes with GFP::TBB-2 and mCherry::H2B. t = 0 seconds corresponds to nuclear envelope breakdown. (PDF) [file pgen.1008751.s001.pdf]

S5 Fig

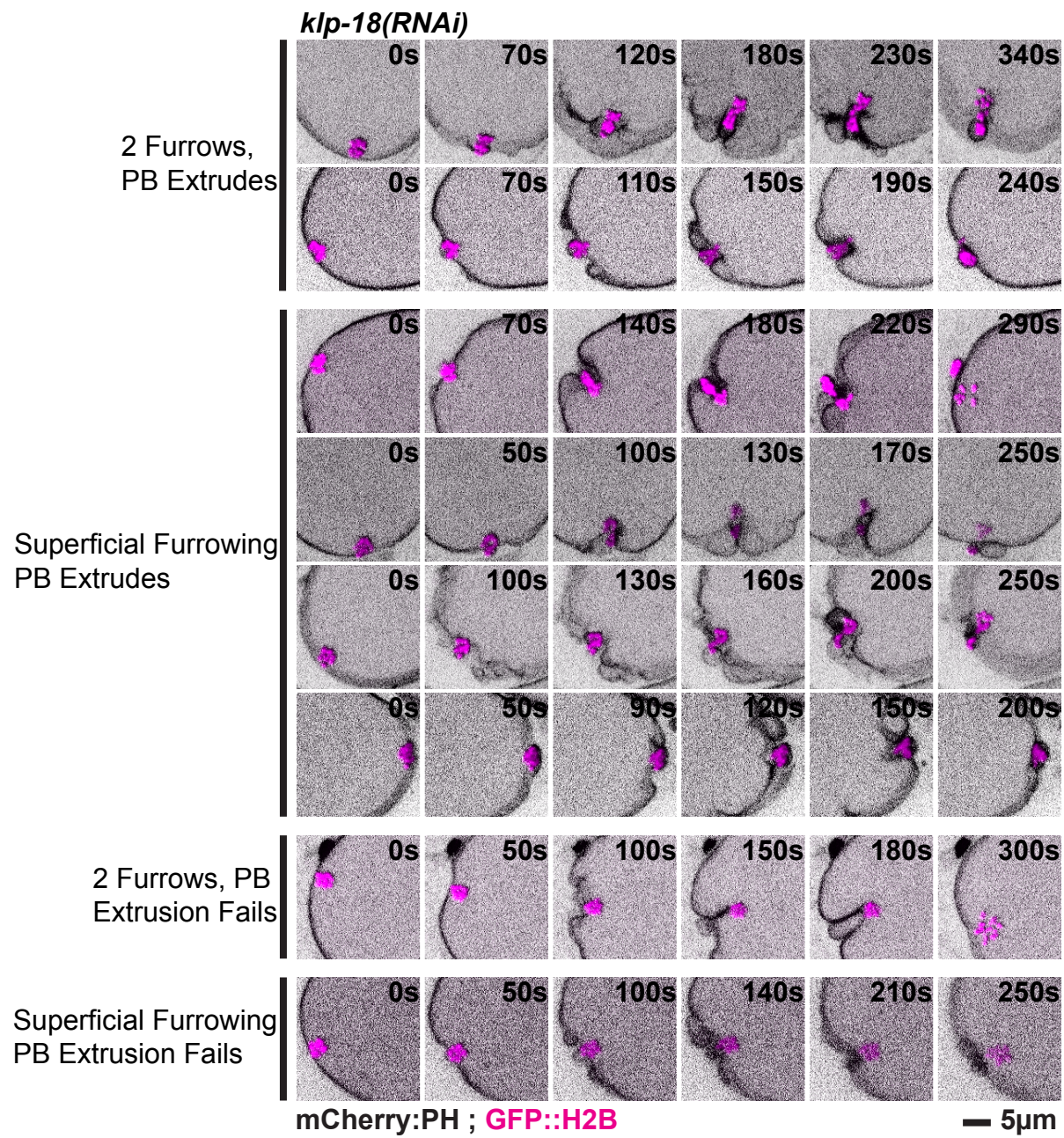

Supplement: S5 Fig — Time-lapse spinning disk confocal images of klp-18(RNAi) oocytes expressing mCherry::PH and GFP::H2B. (PDF) [file pgen.1008751.s005.pdf]

S7 Fig

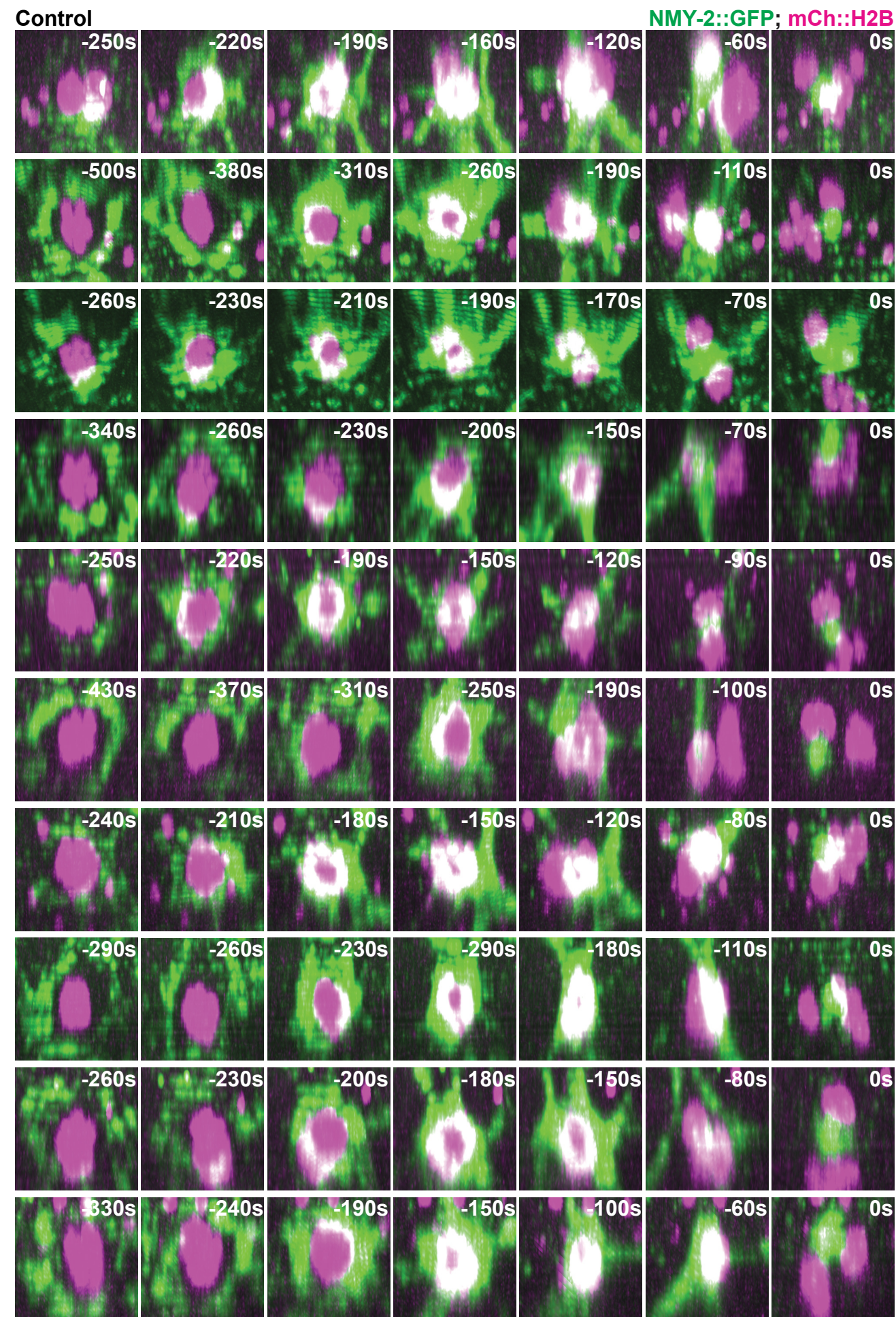

Supplement: S7 Fig — Three-dimensionally projected and rotated spinning disk confocal time-lapse images of control oocytes expressing NMY-2::GFP and mCherry::H2B; t = 0 seconds in this and subsequent Fig 5 related supplements (S8–S10 Figs) corresponds to the end of meiosis I and beginning of meiosis II (see Materials and methods). (PDF) [file pgen.1008751.s007.pdf]

S9 Fig

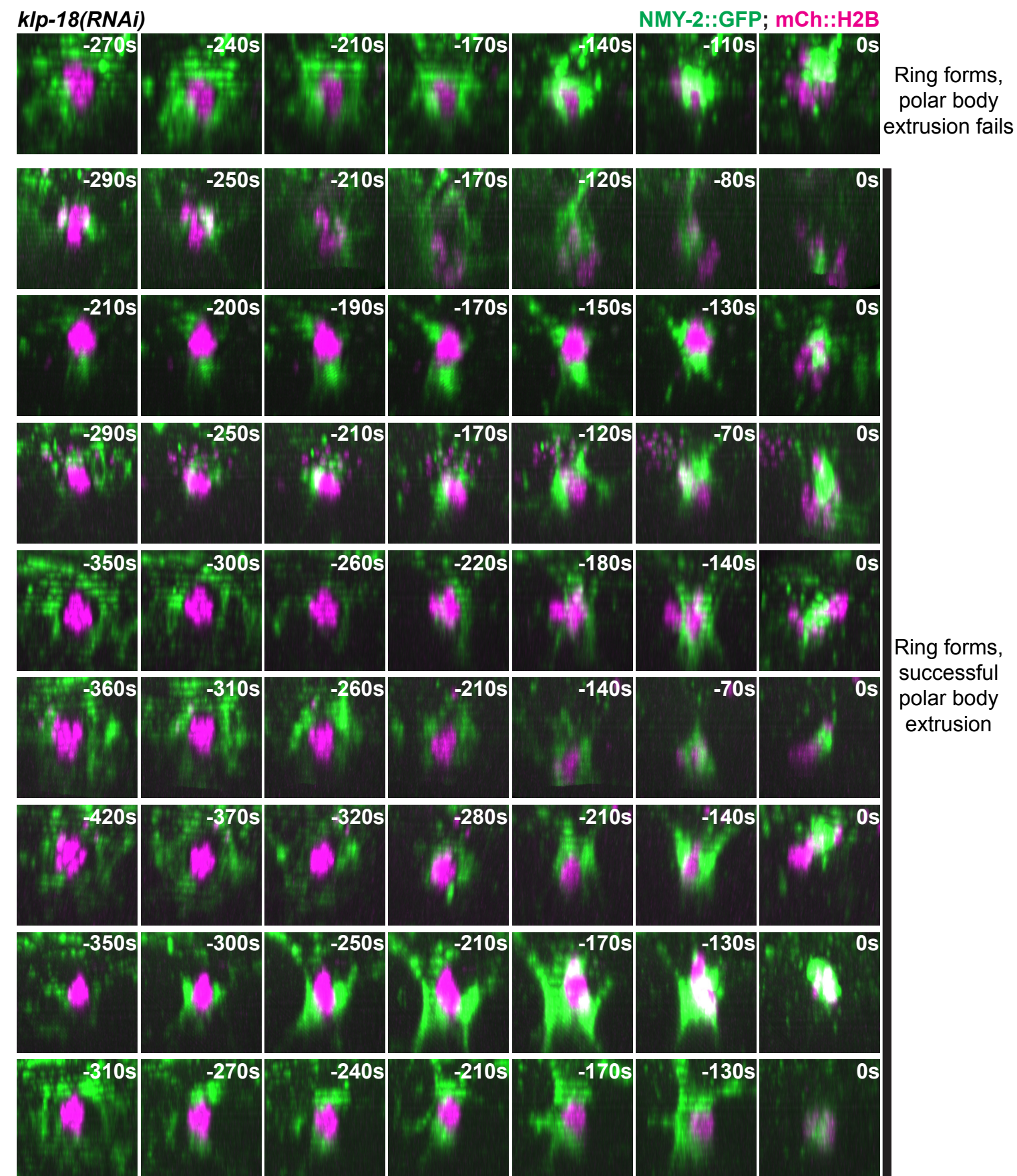

Supplement: S9 Fig — Three-dimensionally projected and rotated spinning disk confocal time-lapse images of klp-18(RNAi) oocytes expressing NMY-2::GFP and mCherry::H2B. (PDF) [file pgen.1008751.s009.pdf]

S11 Fig  
Control  
10μm

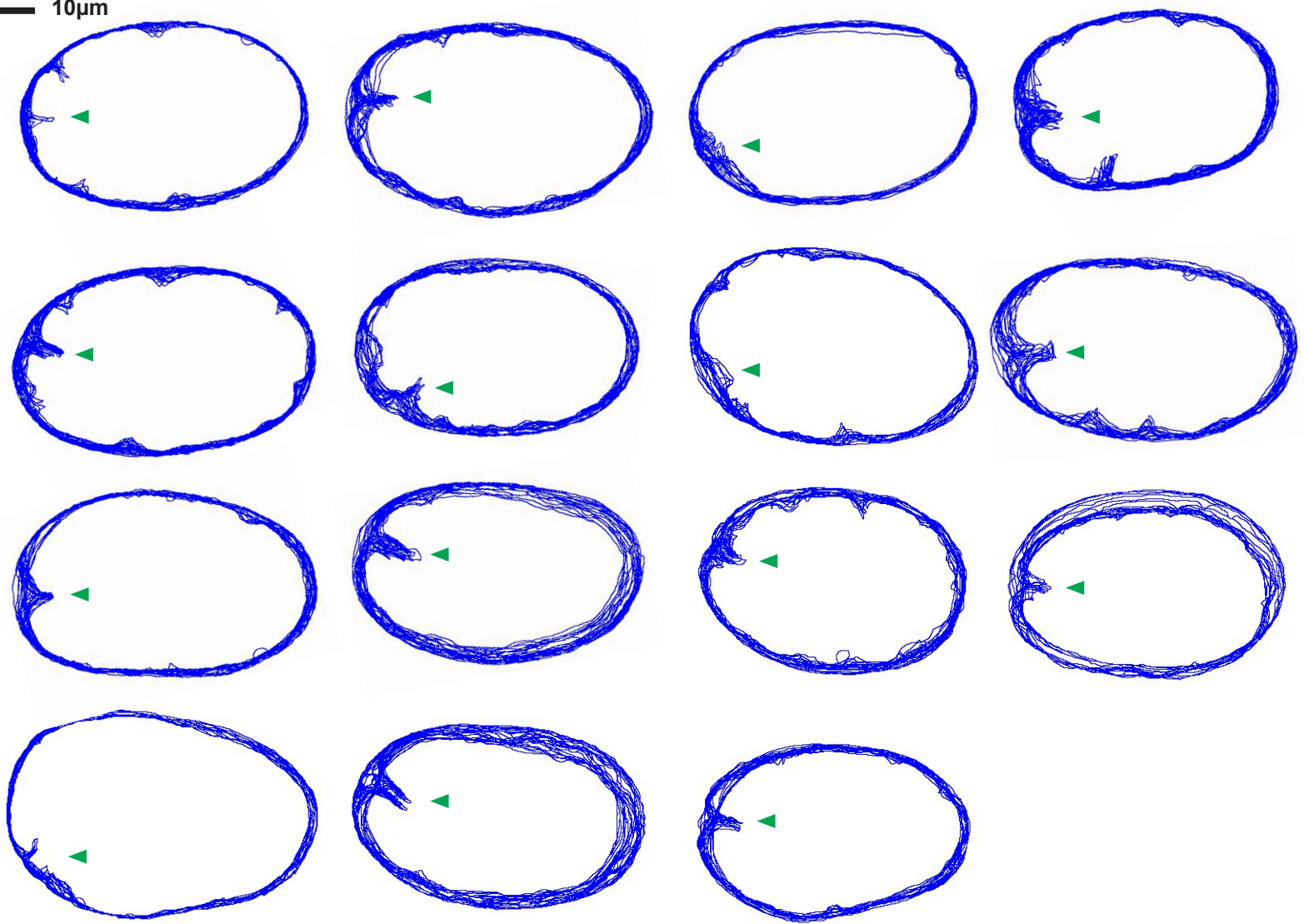

Supplement: S11 Fig — Control oocyte membrane temporal overlays depicting membrane positions over time at a single focal plane throughout meiosis I. Arrowheads indicate approximate location of the meiotic spindle and spindle-associated membrane. (PDF) [file pgen.1008751.s011.pdf]

S12 Fig  
*cls-2(or1948)*  
10μm

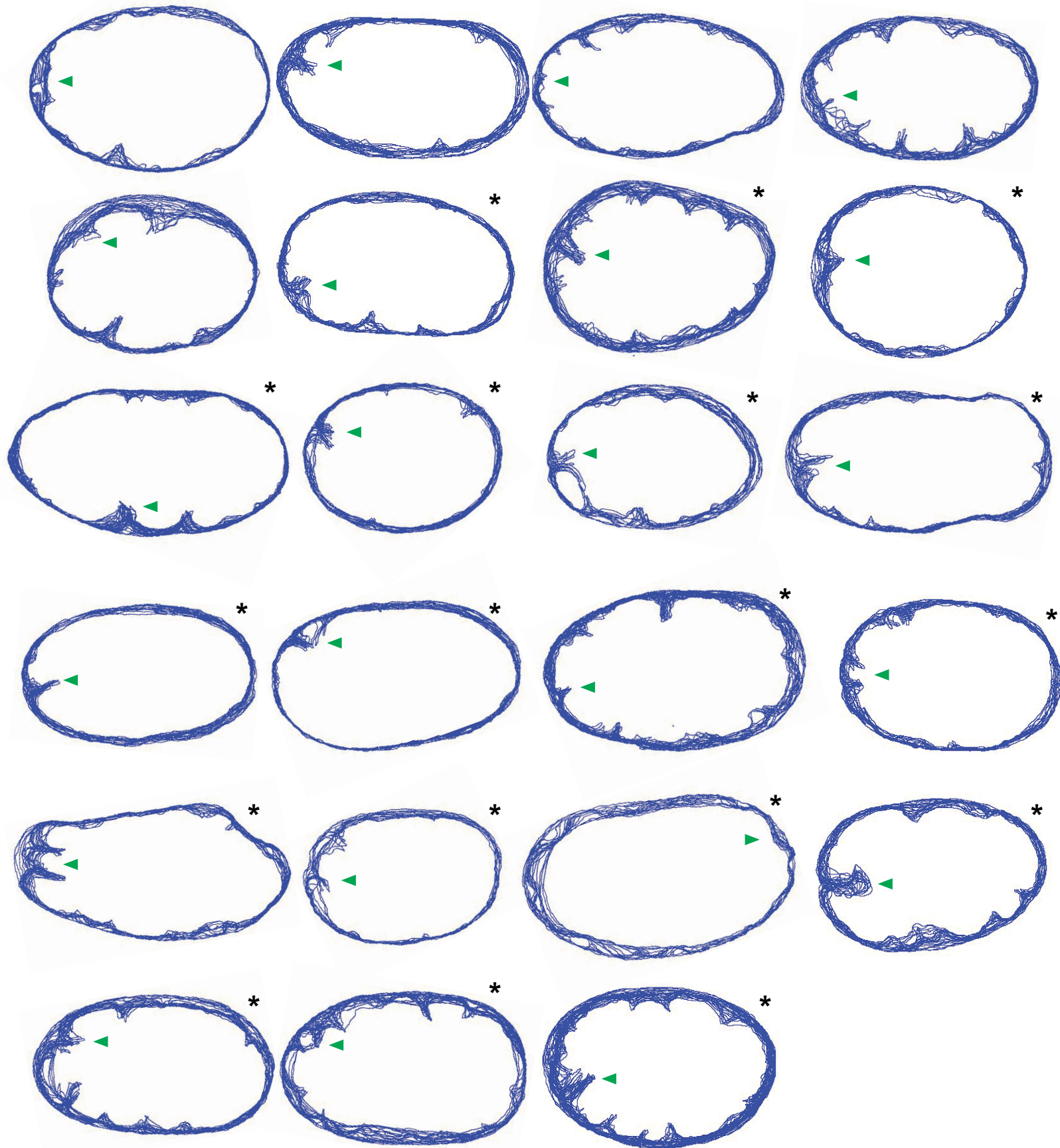

Supplement: S12 Fig — cls-2 mutant oocyte membrane temporal overlays depicting membrane positions over time at a single focal plane throughout meiosis I. Asterisks indicate oocytes in which polar body extrusion failed. Arrowheads indicate approximate location of the meiotic spindle and spindle-associated membrane. (PDF) [file pgen.1008751.s012.pdf]

*klp-18(RNAi)*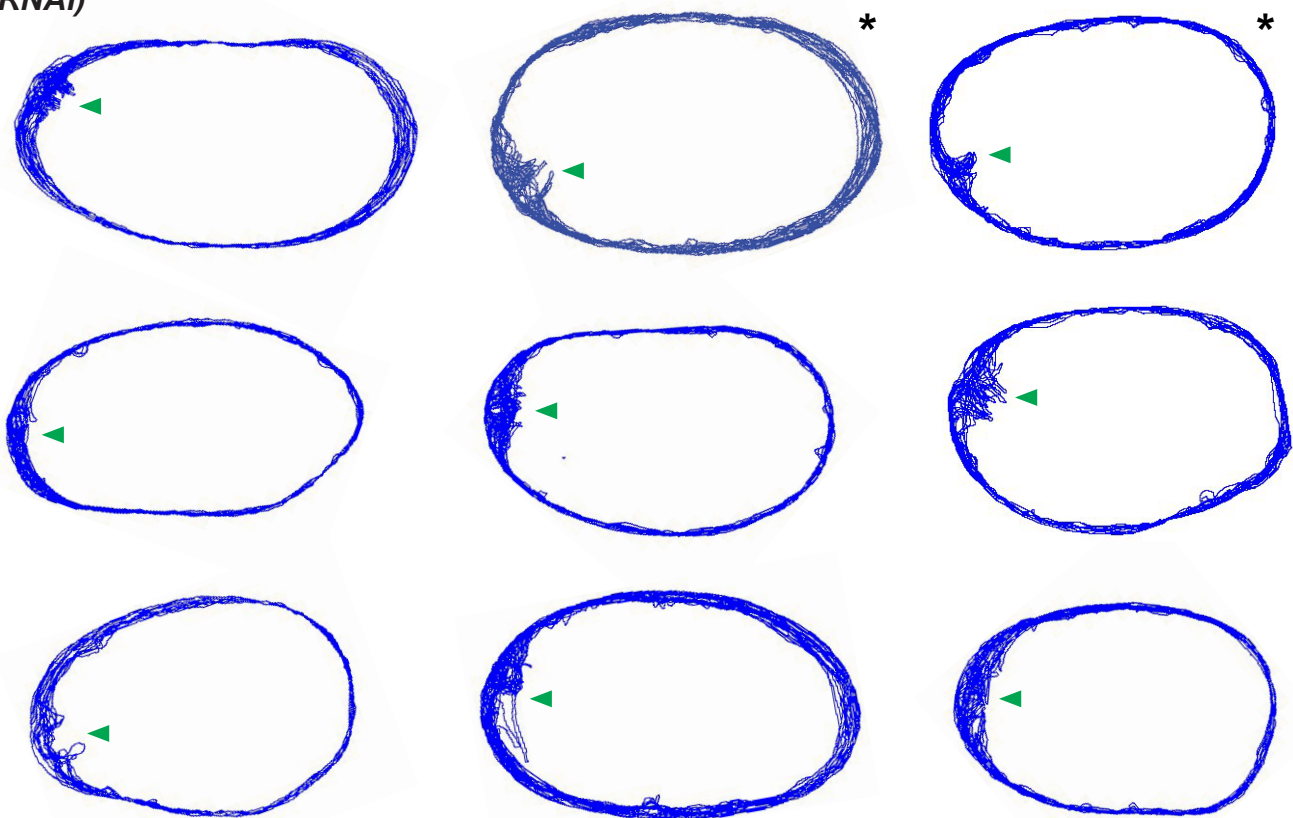*mei-1(RNAi)*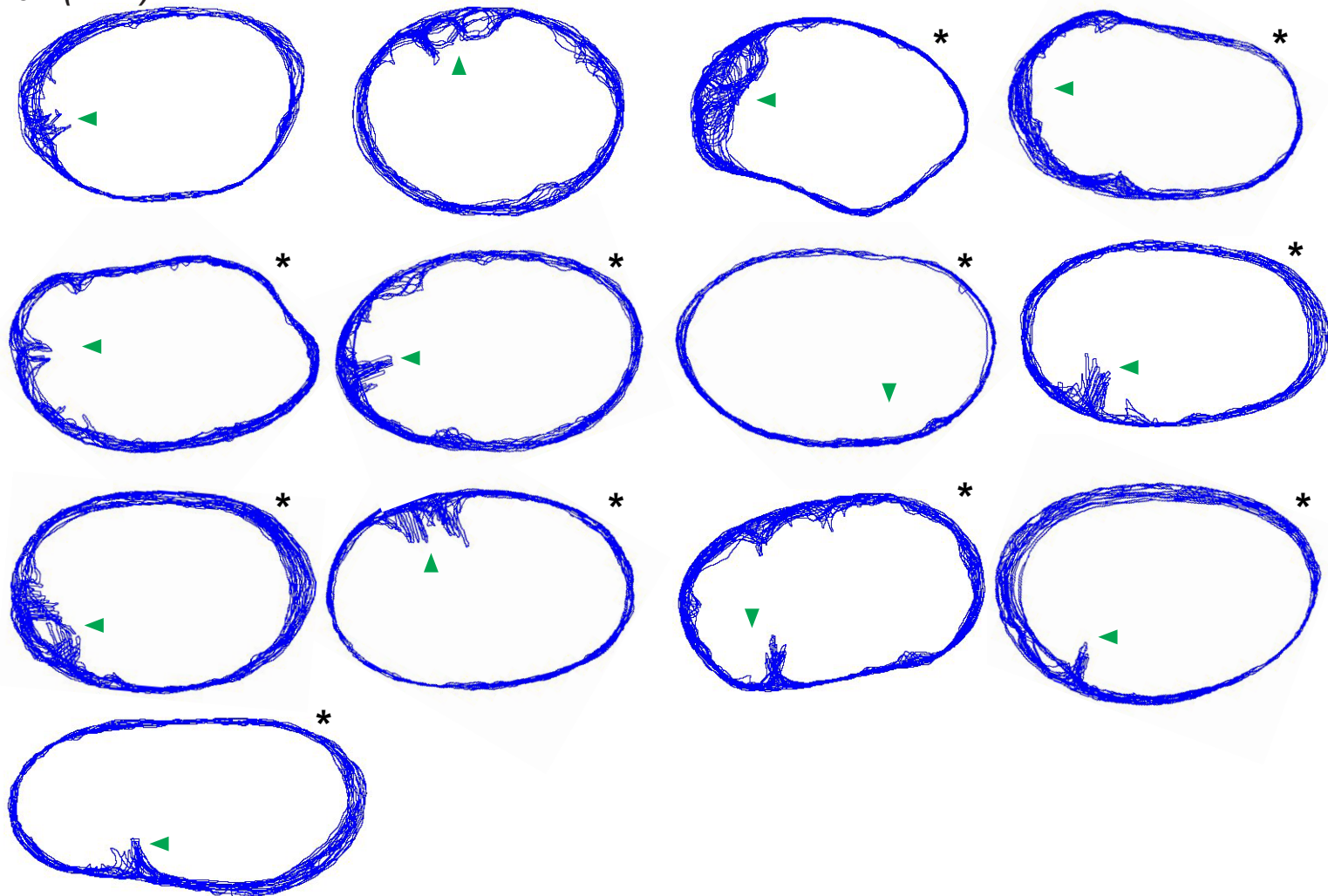

Supplement: S13 Fig — klp-18(RNAi) and mei-1(RNAi) oocyte membrane temporal overlays depicting membrane positions over time at a single focal plane throughout meiosis I. Asterisks indicate oocytes in which polar body extrusion failed. Arrowheads indicate approximate location of the meiotic spindle and spindle-associated membrane. (PDF) [file pgen.1008751.s013.pdf]

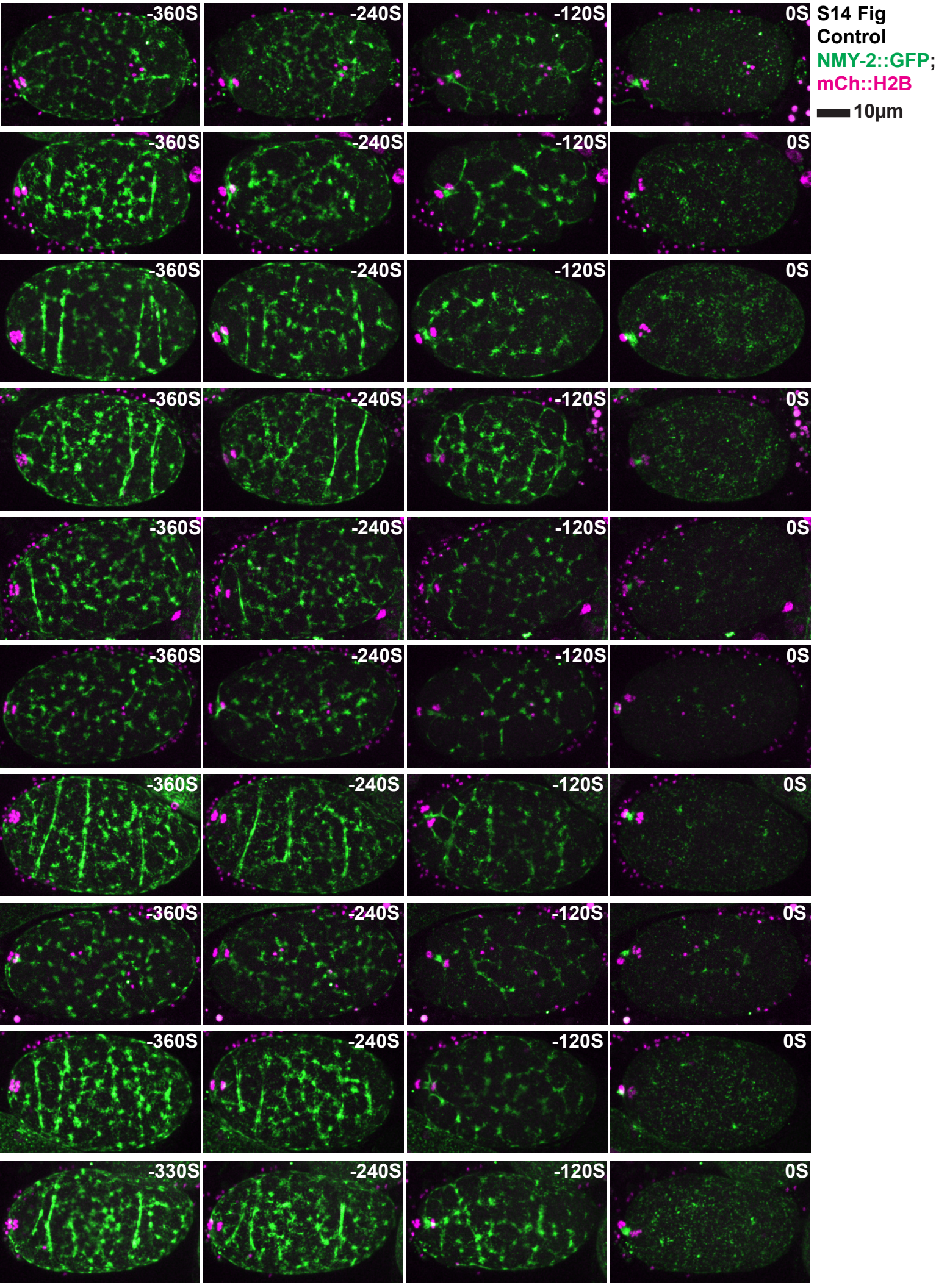

Supplement: S14 Fig — Time-lapse spinning disk confocal images of control oocytes expressing NMY-2::GFP and mCherry:H2B; t = 0 seconds corresponds to the end of meiosis I and beginning of meiosis II in this and subsequent Fig 7 related supplements (S15–S18 Figs). (PDF) [file pgen.1008751.s014.pdf]

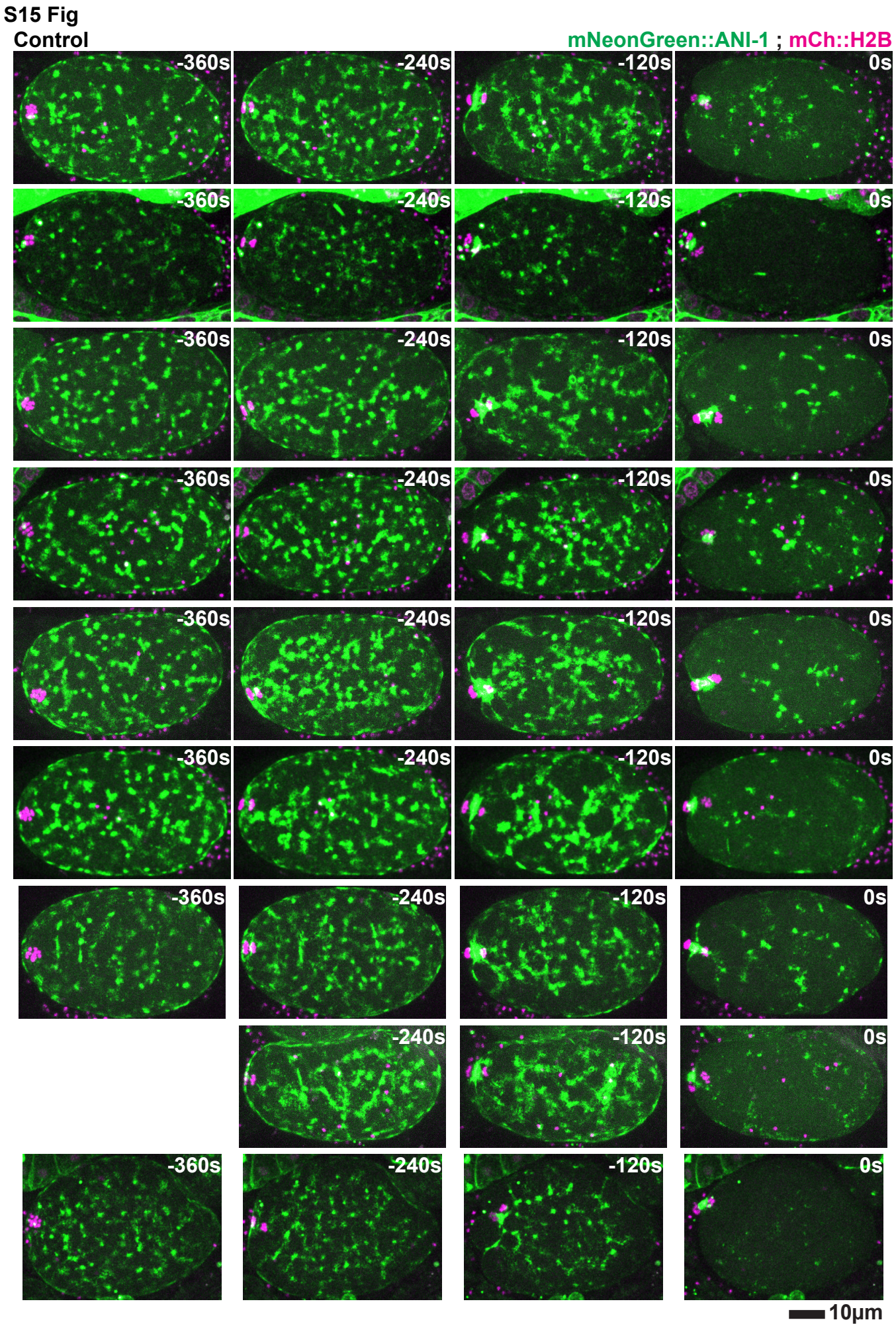

Supplement: S15 Fig — Time-lapse spinning disk confocal images of control oocytes expressing mNeonGreen::ANI-1 and mCherry::H2B. (PDF) [file pgen.1008751.s015.pdf]

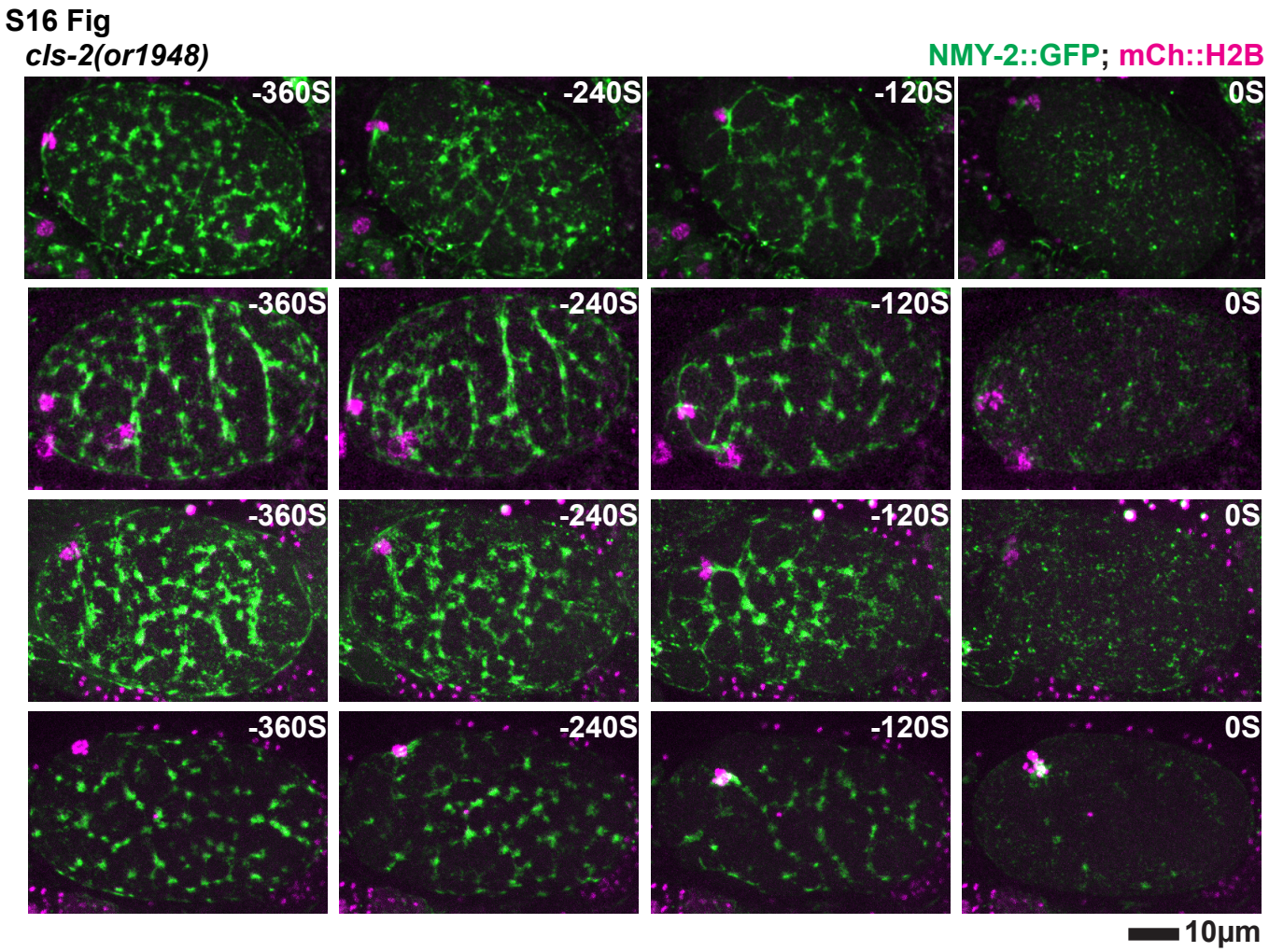

Supplement: S16 Fig — Time-lapse spinning disk confocal images of cls-2 mutant oocytes expressing NMY-2::GFP and mCherry::H2B. All oocytes shown succeeded in polar body extrusion. (PDF) [file pgen.1008751.s016.pdf]

S17 Fig

*cls-2(or1948)*

NMY-2::GFP; mCh::H2B

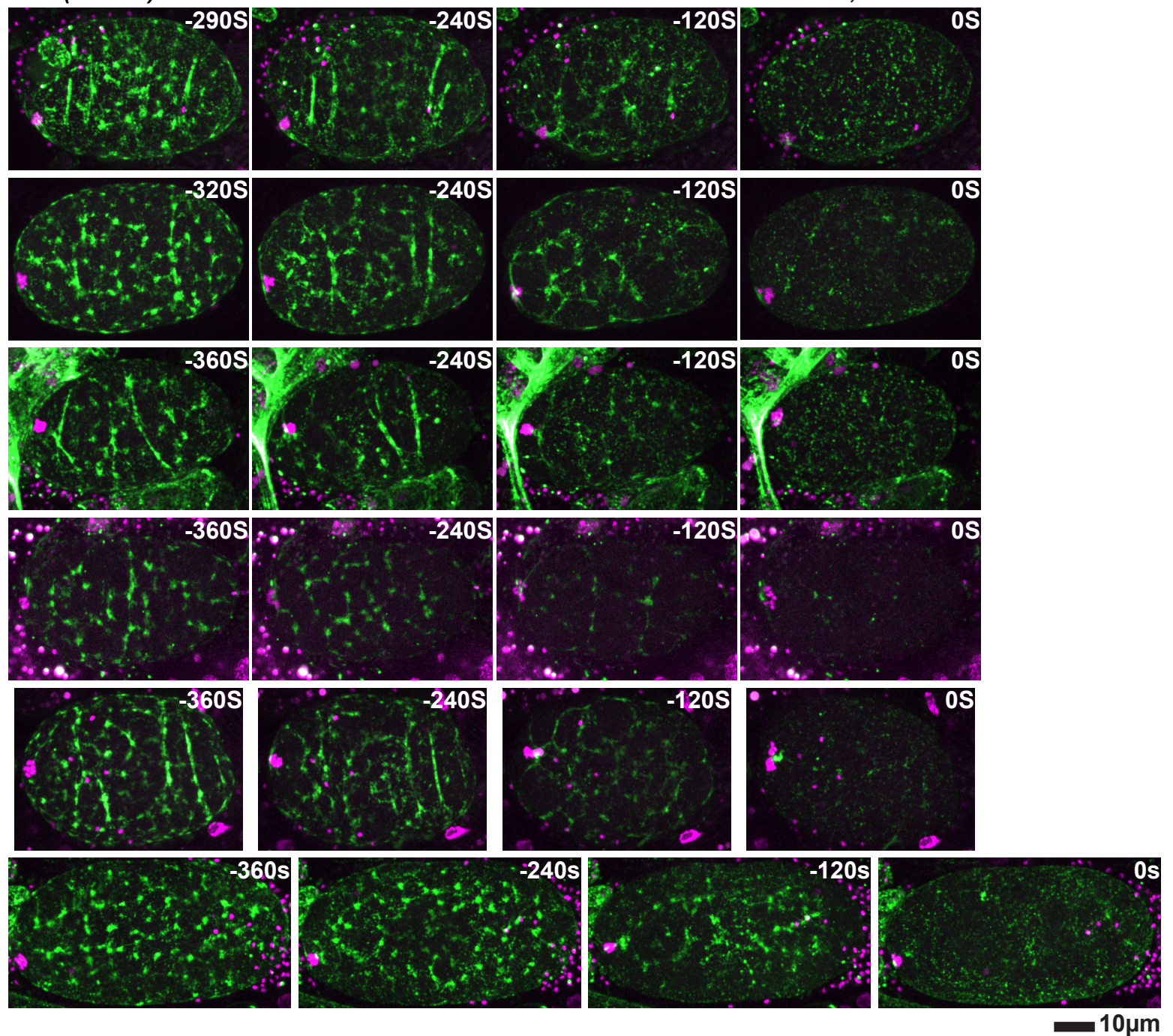

Supplement: S17 Fig — Time-lapse spinning disk confocal images of cls-2 mutant oocytes expressing NMY-2::GFP and mCherry::H2B. All oocytes shown failed in polar body extrusion. (PDF) [file pgen.1008751.s017.pdf]

S18 Fig

*cls-2(or1948)*

mNeonGreen::ANI-1; mCh::H2B

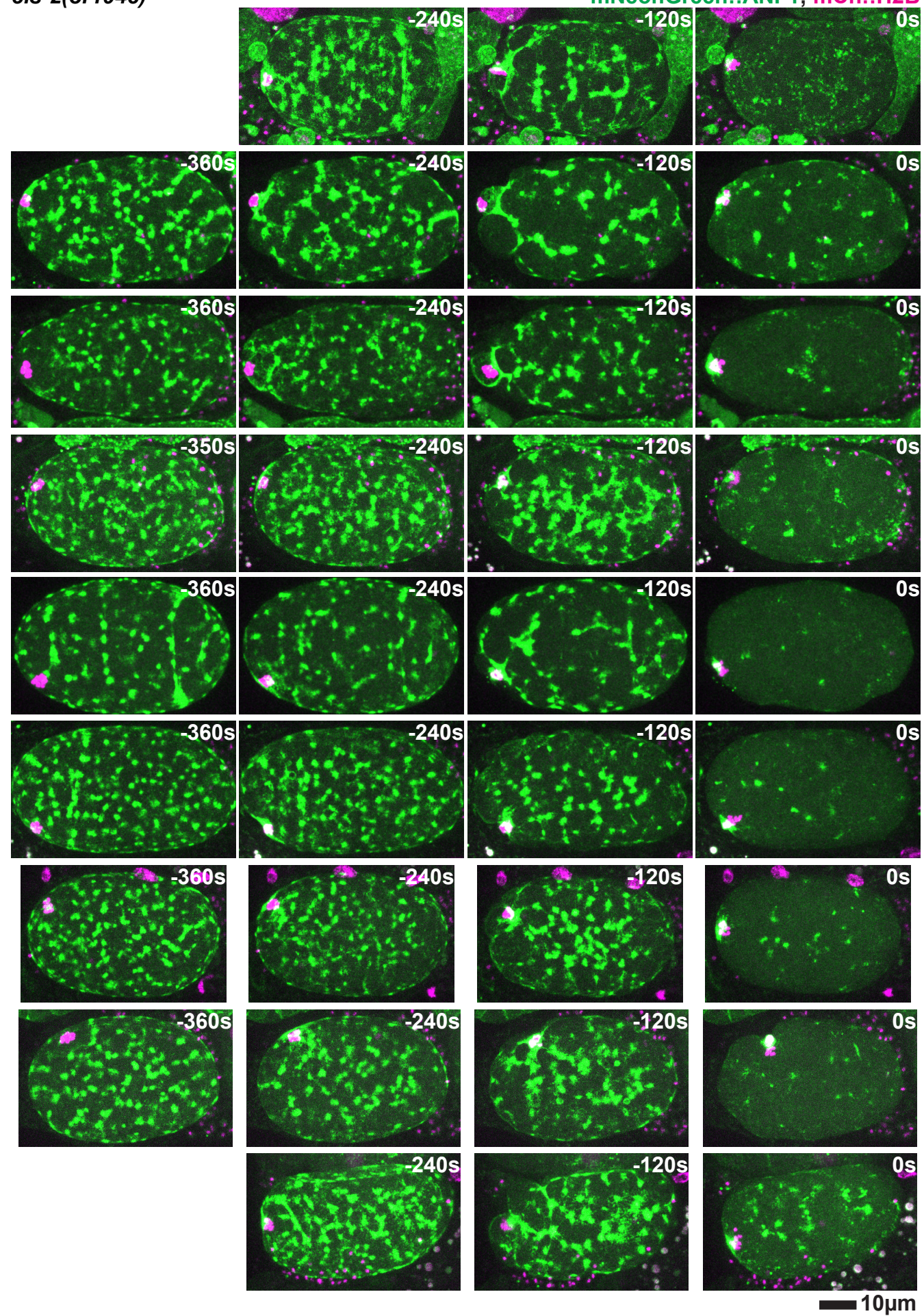

Supplement: S18 Fig — Time-lapse spinning disk confocal images of cls-2 mutant oocytes expressing mNeonGreen::ANI-1 and mCherry::H2B; t = 0s corresponds to the end of meiosis I and beginning of meiosis II. All oocytes shown failed in polar body extrusion. (PDF) [file pgen.1008751.s018.pdf]

S19 Fig

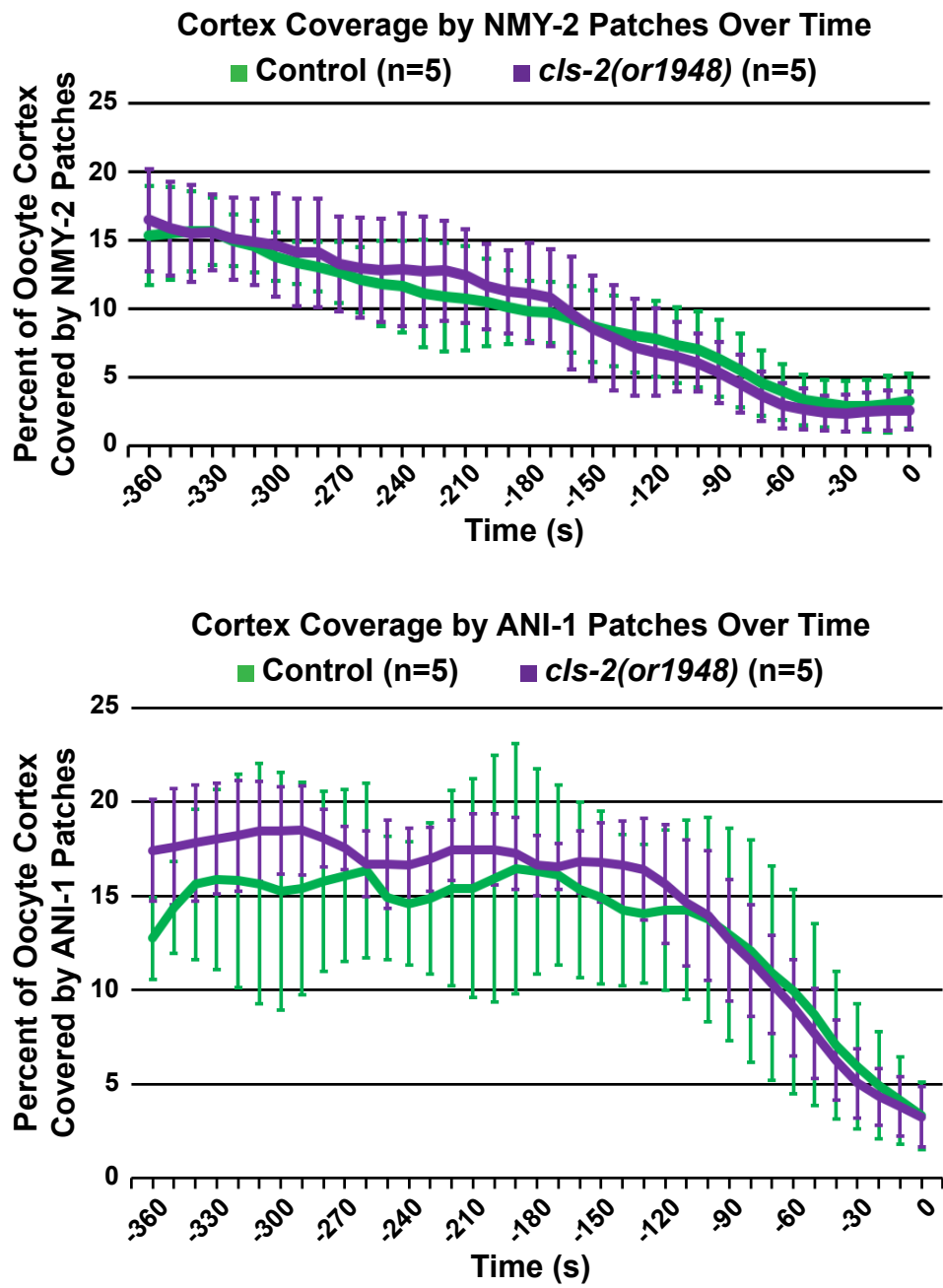

Supplement: S19 Fig — Graphs showing the average percent of the oocyte cortex covered by NMY-2::GFP or mNG::ANI-1 in control or cls-2(or1948) oocytes. Error bars show the standard deviation, and t = 0s corresponds to the end of meiosis I and beginning of meiosis II. (PDF) [file pgen.1008751.s019.pdf]
