# Supplementary material for: C. elegans CLASP/CLS-2 negatively regulates membrane ingression throughout the oocyte cortex and is required for polar body extrusion
Source: PLoS Genet. 2020 Oct 7;16(10):e1008751. doi: 10.1371/journal.pgen.1008751 (PMC7571700; doi:10.1371/journal.pgen.1008751)
Supplement: S2 Fig — Time-lapse spinning disk confocal images of control oocytes expressing mCherry::PH and GFP::H2B; t = 0 seconds here and in subsequent Fig 4 related supplements (S3–S6 Figs) corresponds to the time point immediately before global cortical furrowing begins, unless otherwise stated. (PDF) [file pgen.1008751.s002.pdf]

S2 Fig

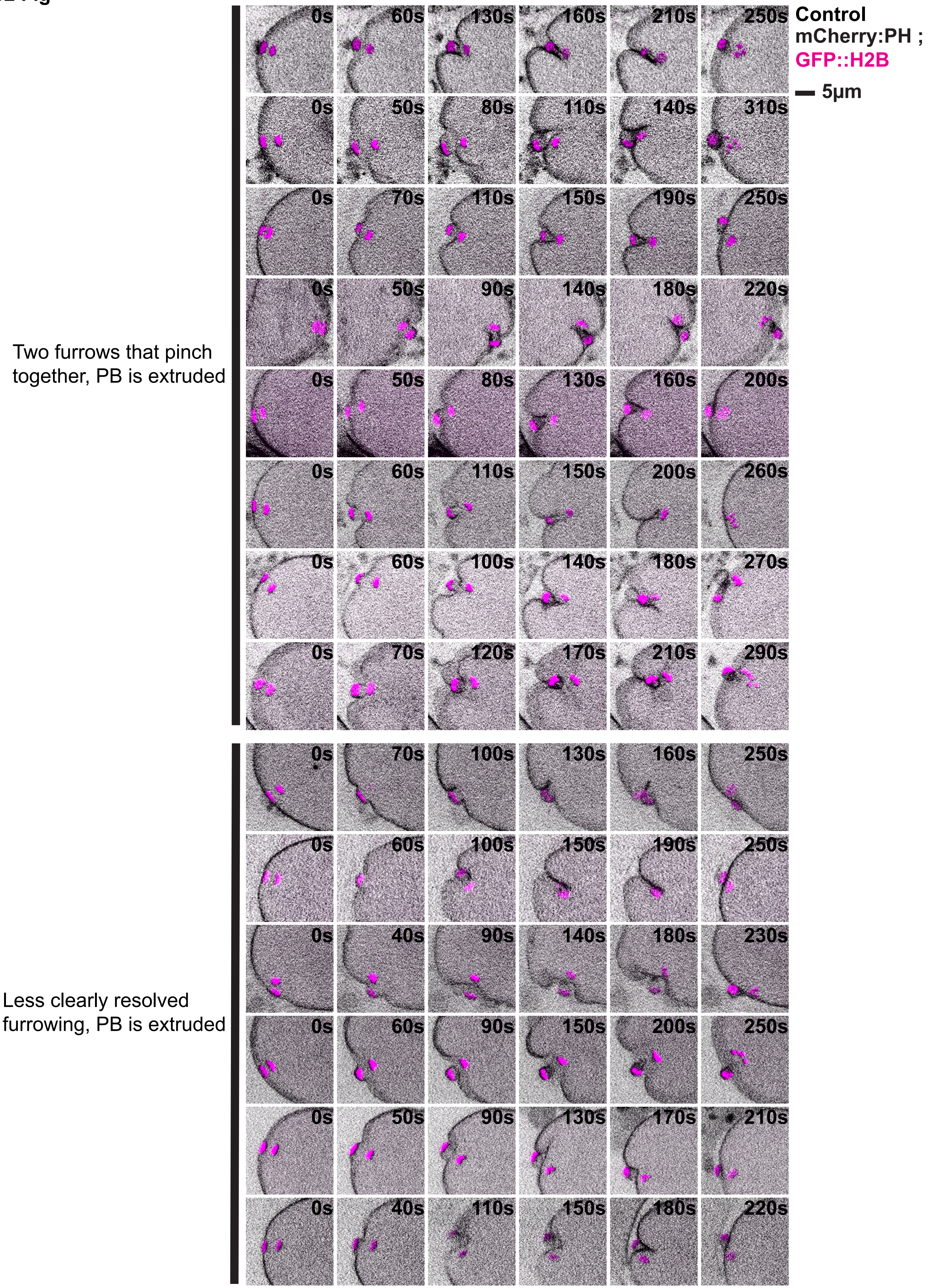
Control
mCherry:PH ;
GFP::H2B
5μm
Two furrows that pinch together, PB is extruded
0s 60s 130s 160s 210s 250s
0s 50s 80s 110s 140s 310s
0s 70s 110s 150s 190s 250s
0s 50s 90s 140s 180s 220s
0s 50s 80s 130s 160s 200s
0s 60s 110s 150s 200s 260s
0s 60s 100s 140s 180s 270s
0s 70s 120s 170s 210s 290s
Less clearly resolved furrowing, PB is extruded
0s 70s 100s 130s 160s 250s
0s 60s 100s 150s 190s 250s
0s 40s 90s 140s 180s 230s
0s 60s 90s 150s 200s 250s
0s 50s 90s 130s 170s 210s
0s 40s 110s 150s 180s 220s
