# Supplementary material for: C. elegans CLASP/CLS-2 negatively regulates membrane ingression throughout the oocyte cortex and is required for polar body extrusion
Source: PLoS Genet. 2020 Oct 7;16(10):e1008751. doi: 10.1371/journal.pgen.1008751 (PMC7571700; doi:10.1371/journal.pgen.1008751)
Supplement: S3 Fig — Time-lapse spinning disk confocal images of cls-2 mutant oocytes expressing mCherry::PH and GFP::H2B. (PDF) [file pgen.1008751.s003.pdf]

S3 Fig

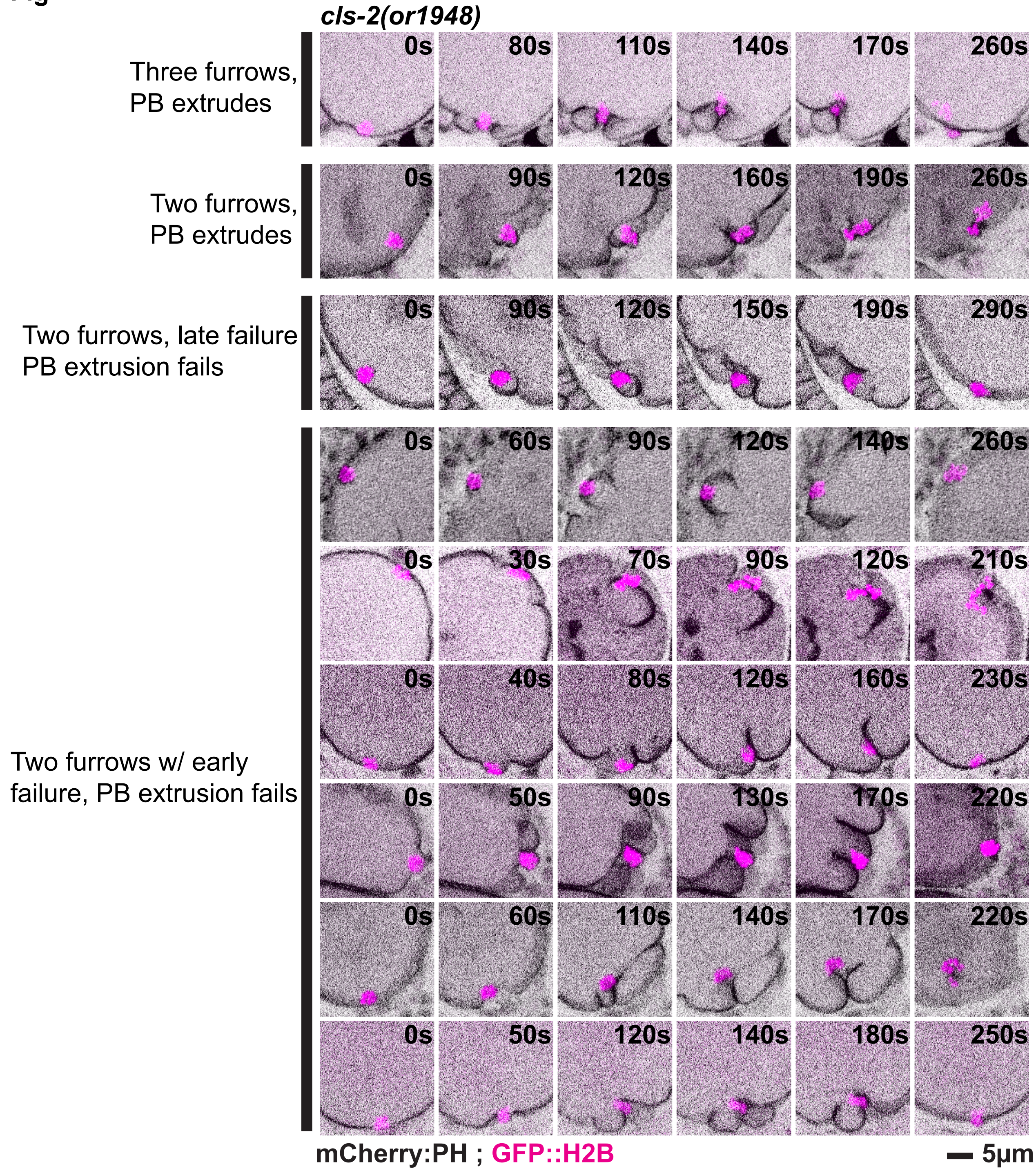
cls-2(or1948)
Three furrows, PB extrudes
0s
80s
110s
140s
170s
260s
Two furrows, PB extrudes
0s
90s
120s
160s
190s
260s
Two furrows, late failure PB extrusion fails
0s
90s
120s
150s
190s
290s
Two furrows w/ early failure, PB extrusion fails
0s
60s
90s
120s
140s
260s
0s
30s
70s
90s
120s
210s
0s
40s
80s
120s
160s
230s
0s
50s
90s
130s
170s
220s
0s
60s
110s
140s
170s
220s
0s
50s
120s
140s
180s
250s
mCherry:PH ; GFP::H2B
5μm
