# Supplementary material for: C. elegans CLASP/CLS-2 negatively regulates membrane ingression throughout the oocyte cortex and is required for polar body extrusion
Source: PLoS Genet. 2020 Oct 7;16(10):e1008751. doi: 10.1371/journal.pgen.1008751 (PMC7571700; doi:10.1371/journal.pgen.1008751)
Supplement: S4 Fig — Time-lapse spinning disk confocal images of cls-2 mutant oocytes expressing mCherry::PH and GFP::H2B. Note that in a small number of cls-2(-) oocytes, we sometimes observe some separation of chromosomes. When examined in conjunction with the mCherry::PH membrane marker, such late separation of chromosomes was always associated with ingression of an oocyte meiotic cleavage furrow that appeared to push chromosomes apart (see rows 4 and 6). (PDF) [file pgen.1008751.s004.pdf]

S4 Fig

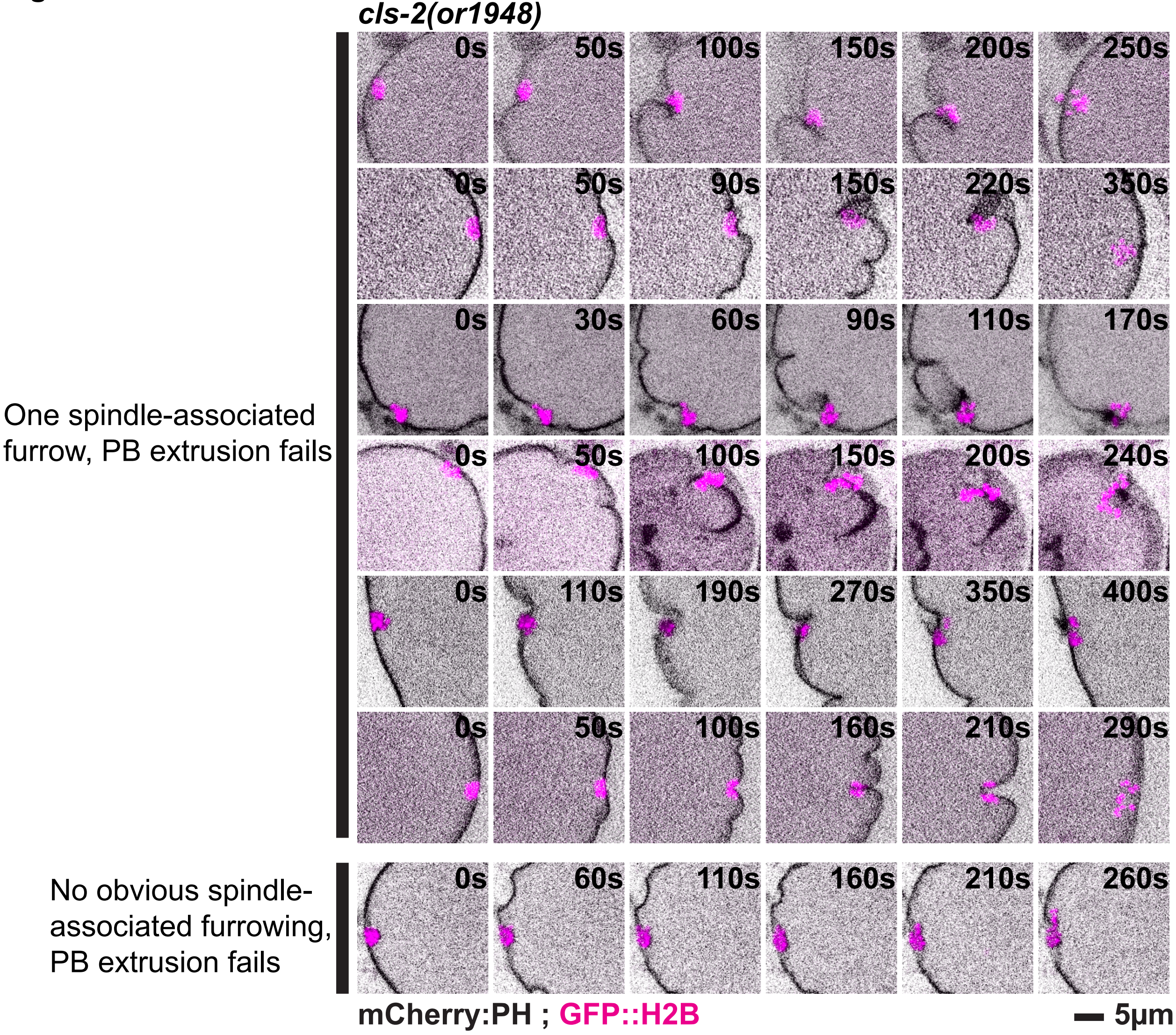
cls-2(or1948)
0s
50s
100s
150s
200s
250s
0s
50s
90s
150s
220s
350s
0s
30s
60s
90s
110s
170s
0s
50s
100s
150s
200s
240s
0s
110s
190s
270s
350s
400s
0s
50s
100s
160s
210s
290s
0s
60s
110s
160s
210s
260s
One spindle-associated furrow, PB extrusion fails
No obvious spindle-associated furrowing, PB extrusion fails
mCherry:PH ; GFP::H2B
5µm
