# Supplementary material for: C. elegans CLASP/CLS-2 negatively regulates membrane ingression throughout the oocyte cortex and is required for polar body extrusion
Source: PLoS Genet. 2020 Oct 7;16(10):e1008751. doi: 10.1371/journal.pgen.1008751 (PMC7571700; doi:10.1371/journal.pgen.1008751)
Supplement: S6 Fig — Time-lapse spinning disk confocal images of mei-1(RNAi) oocytes expressing mCherry::PH and GFP::H2B. (PDF) [file pgen.1008751.s006.pdf]

S6 Fig

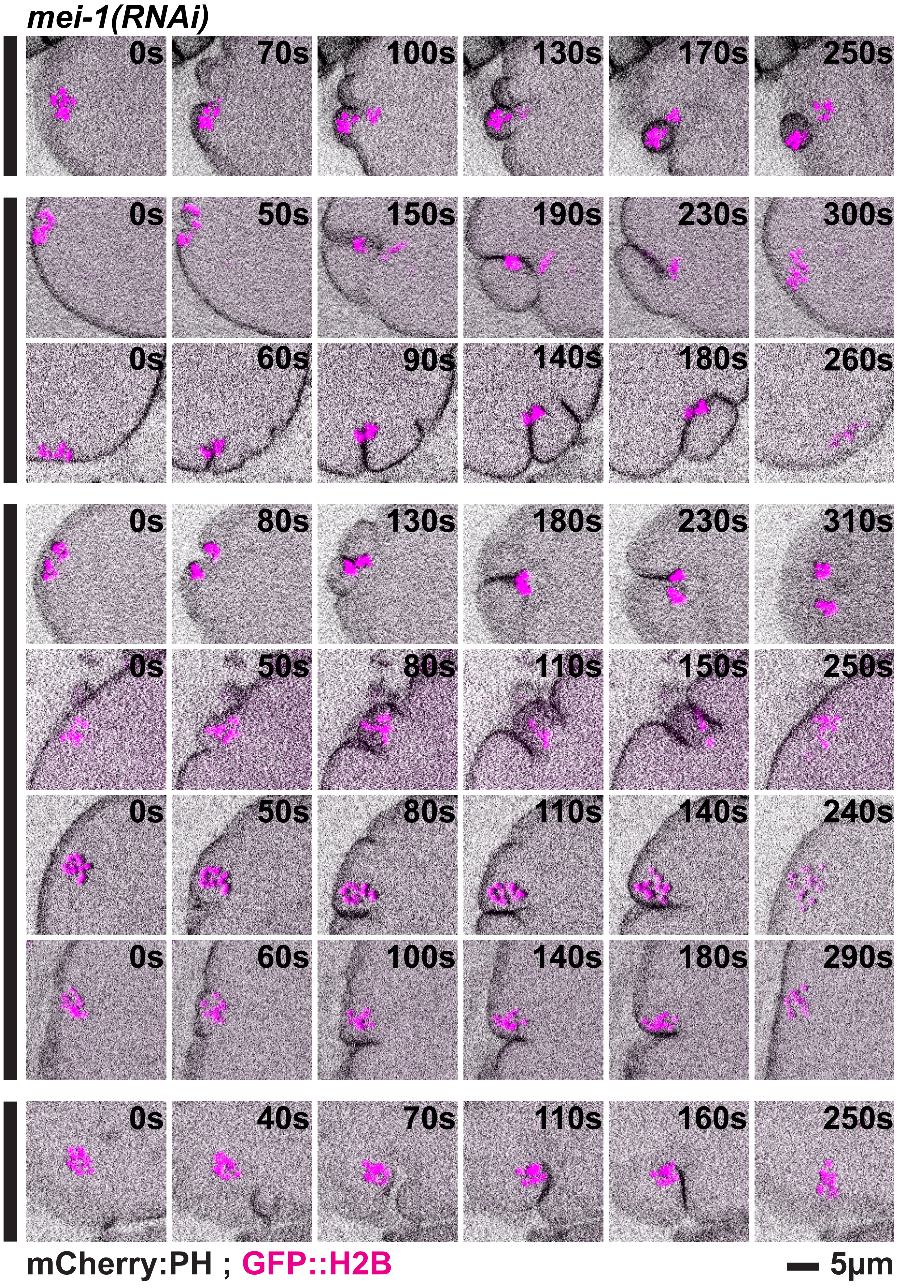
mei-1(RNAi)
Two furrows, PB extrudes
0s 70s 100s 130s 170s 250s
Two furrows w/ late failure, PB extrusion fails
0s 50s 150s 190s 230s 300s
0s 60s 90s 140s 180s 260s
Two furrows w/ early failure, PB extrusion fails
0s 80s 130s 180s 230s 310s
0s 50s 80s 110s 150s 250s
0s 50s 80s 110s 140s 240s
0s 60s 100s 140s 180s 290s
One furrow, PB extrusion fails
0s 40s 70s 110s 160s 250s
mCherry:PH ; GFP::H2B
5μm
