# Supplementary material for: C. elegans CLASP/CLS-2 negatively regulates membrane ingression throughout the oocyte cortex and is required for polar body extrusion
Source: PLoS Genet. 2020 Oct 7;16(10):e1008751. doi: 10.1371/journal.pgen.1008751 (PMC7571700; doi:10.1371/journal.pgen.1008751)
Supplement: S8 Fig — Three-dimensionally projected and rotated spinning disk confocal time-lapse images of cls-2 mutant oocytes expressing NMY-2::GFP and mCherry::H2B. (PDF) [file pgen.1008751.s008.pdf]

S8 Fig

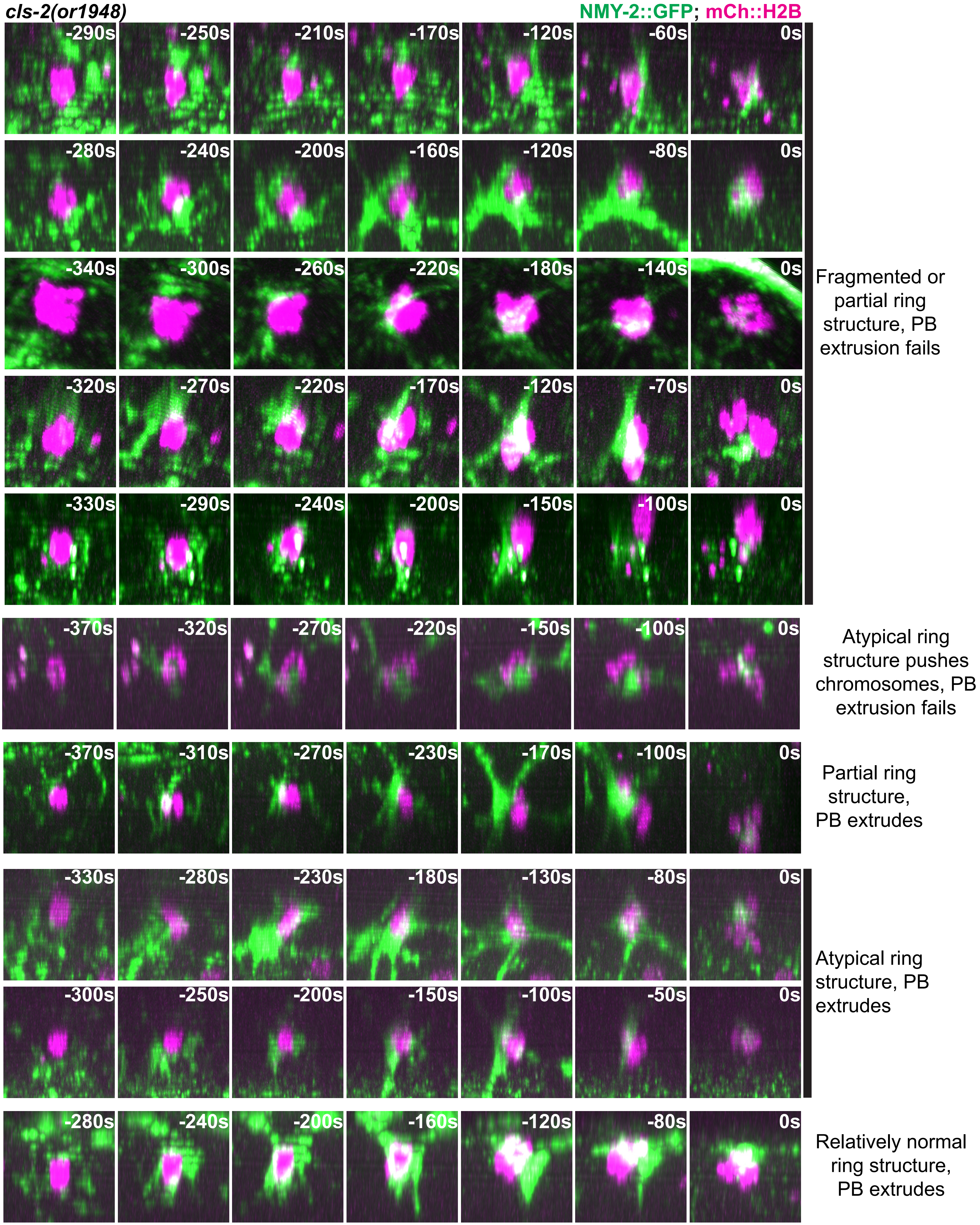
cls-2(or1948)
NMY-2::GFP; mCh::H2B
-290s
-250s
-210s
-170s
-120s
-60s
0s
-280s
-240s
-200s
-160s
-120s
-80s
0s
-340s
-300s
-260s
-220s
-180s
-140s
0s
-320s
-270s
-220s
-170s
-120s
-70s
0s
-330s
-290s
-240s
-200s
-150s
-100s
0s
Fragmented or partial ring structure, PB extrusion fails
-370s
-320s
-270s
-220s
-150s
-100s
0s
Atypical ring structure pushes chromosomes, PB extrusion fails
-370s
-310s
-270s
-230s
-170s
-100s
0s
Partial ring structure, PB extrudes
-330s
-280s
-230s
-180s
-130s
-80s
0s
-300s
-250s
-200s
-150s
-100s
-50s
0s
Atypical ring structure, PB extrudes
-280s
-240s
-200s
-160s
-120s
-80s
0s
Relatively normal ring structure, PB extrudes
