# Supplementary material for: C. elegans CLASP/CLS-2 negatively regulates membrane ingression throughout the oocyte cortex and is required for polar body extrusion
Source: PLoS Genet. 2020 Oct 7;16(10):e1008751. doi: 10.1371/journal.pgen.1008751 (PMC7571700; doi:10.1371/journal.pgen.1008751)
Supplement: S10 Fig — Three-dimensionally projected and rotated spinning disk confocal time-lapse images of mei-1(RNAi) oocytes expressing NMY-2::GFP and mCherry::H2B. (PDF) [file pgen.1008751.s010.pdf]

S10 Fig

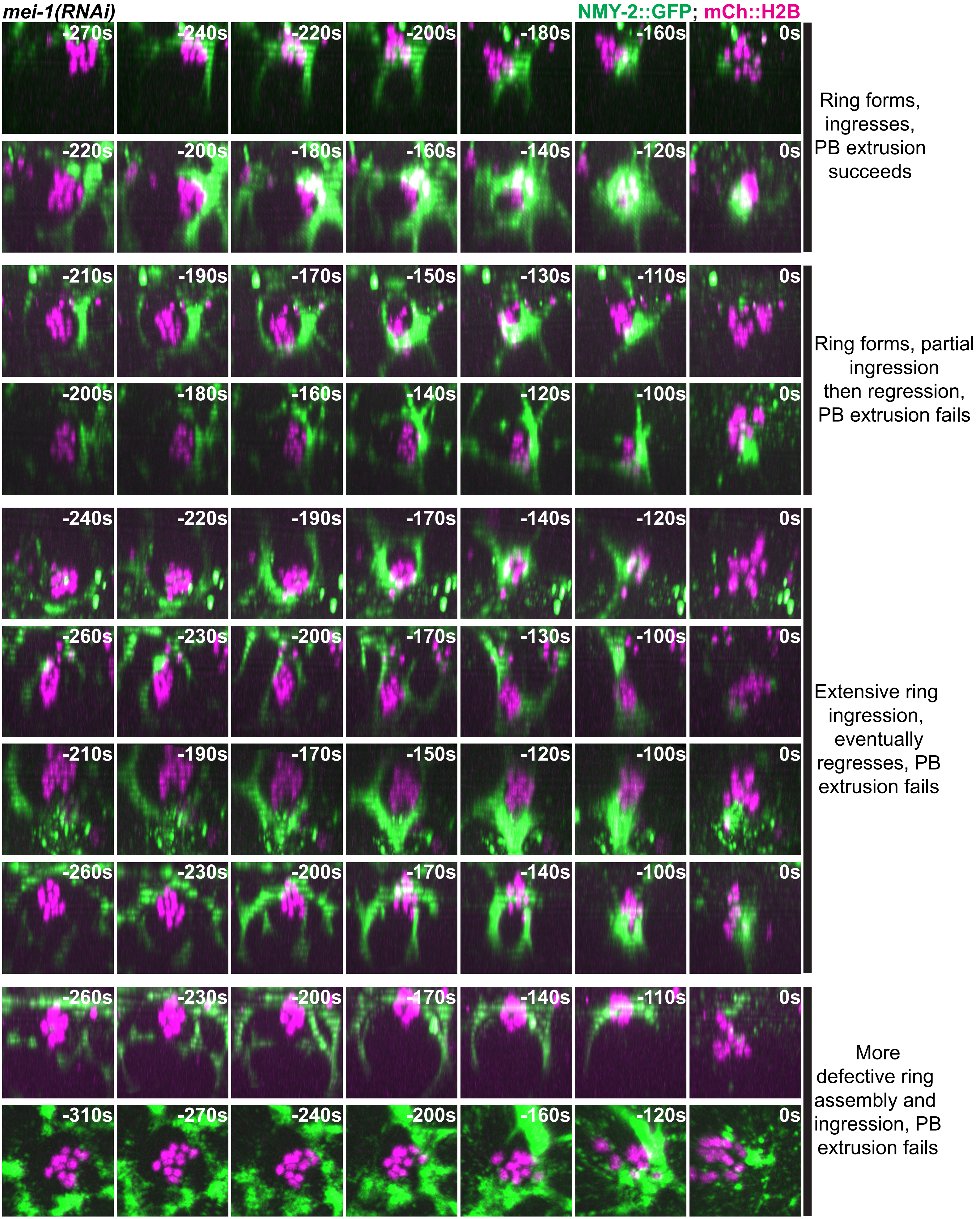

Not shown - 1 example of ring closure failure, and 1 example of successful ring formation, ingression, and PBE
