# Supplementary material for: C. elegans CLASP/CLS-2 negatively regulates membrane ingression throughout the oocyte cortex and is required for polar body extrusion
Source: PLoS Genet. 2020 Oct 7;16(10):e1008751. doi: 10.1371/journal.pgen.1008751 (PMC7571700; doi:10.1371/journal.pgen.1008751)
Supplement: S1 Table — (PDF) [file pgen.1008751.s046.pdf]

Supplemental Table 1 - *C. elegans* strains used in this study

Strain #	Genotype
N2	*Wild type*
EU3020	*cls-2(or1948) /qC1[qIs26] III*
EU3021	*cls-2(or1949) /qC1[qIs26] III*
EU3022	*cls-2(or1950) /qC1[qIs26] III*
EU3023	*cls-2(or1951) /qC1[qIs26] III*
EU3030	*ijmSi3 [pJD342/pJD330; ChrI_5'mex-5_cls-2reenc::GFP_3'tbb-2; cb-unc-119(+)] I; unc-119(ed3) III?; itIs37[pie-1p::mCherry::H2B::pie-1 3'UTR + unc-119(+)] IV*
EU2942	*ruIs57[pie-1p::GFP::tubulin + unc-119(+)]; itIs37[pie-1p::mCherry::H2B::pie-1 3'UTR + unc-119(+)] IV*
EU3025	*cls-2(or1948) /qC1[qIs26] III ; ruIs57[pie-1p::GFP::tubulin + unc-119(+)]; itIs37[pie-1p::mCherry::H2B::pie-1 3'UTR + unc-119(+)] IV*
EU2876	*or1935[GFP::aspm-1] I; itIs37[pie-1p::mCherry::H2B::pie-1 3'UTR + unc-119(+)] IV*
EU3031	*or1935[GFP::aspm-1] I ; cls-2(or1948)/qC1[qIs26] III ; itIs37[pie-1p::mCherry::H2B::pie-1 3'UTR + unc-119(+)] IV*
OD224	*unc-119(ed3) III; ltIs37 [pAA64; pie-1/mCHERRY::his-58; unc-119(+)] ltIs14 [pASM05; pie-1/GFP-TEV-STag::air-2; unc-119(+)]IV*
EU3108	*cls-2(or1948) /qC1[qIs26] III; ltIs37 [pAA64; pie-1/mCHERRY::his-58; unc-119(+)] ltIs14 [pASM05; pie-1/GFP-TEV-STag::air-2; unc-119(+)]IV*
EU3061	*mgSi43 [cyk-4p::cyk-4::GFP::pie-1 3'UTR + Cbr-unc-119(+)] II ; itIs37[pie-1p::mCherry::H2B::pie-1 3'UTR + unc-119(+)] IV*
EU3067	*cls-2(or1948) /qC1[qIs26] III ; mgSi43 [cyk-4p::cyk-4::GFP::pie-1 3'UTR + Cbr-unc-119(+)] II ; itIs37[pie-1p::mCherry::H2B::pie-1 3'UTR + unc-119(+)] IV*
EU3122	*ruIs32[unc-119(+) pie-1::GFP::H2B] (may contain unc-119 (eds) III; ltIs44V[pie-1p-mCherry::PH(PLC1delta1) + unc-119(+)]*
EU3105	*cls-2(or1948) /qC1[qIs26] ruIs32[unc-119(+) pie-1::GFP::H2B] (may contain unc-119 (eds) III; ltIs44V[pie-1p-mCherry::PH(PLC1delta1) + unc-119(+)]*
EU3080	*nmy-2(cp13[nmy-2::gfp + LoxP]) I; itIs37[pie-1p::mCherry::H2B::pie-1 3'UTR + unc-119(+)] IV*
EU3079	*nmy-2(cp13[nmy-2::gfp + LoxP]) I; cls-2(or1948) /qC1[qIs26] III; itIs37[pie-1p::mCherry::H2B::pie-1 3'UTR + unc-119(+)] IV*
EU3146	*ani-1(mon7[mNeonGreen^3xFlag::ani-1]) III; itIs37[pie-1p::mCherry::H2B::pie-1 3'UTR + unc-119(+)] IV*
EU3153	*cls-2(or1948) /qC1[qIs26] ani-1(mon7[mNeonGreen^3xFlag::ani-1]) III; itIs37[pie-1p::mCherry::H2B::pie-1 3'UTR + unc-119(+)] IV*
EU3166	*nmy-2(cp52[nmy-2::mkate2 + LoxP unc-119(+) LoxP]) I; ani-1(mon7[mNeonGreen^3xFlag::ani-1]) III; itIs37[pie-1p::mCherry::H2B::pie-1 3'UTR + unc-119(+)] IV*
EU3172	*nmy-2(cp52[nmy-2::mkate2 + LoxP unc-119(+) LoxP]) I; cls-2(or1948)/qC1[qIs26] ani-1(mon7[mNeonGreen^3xFlag::ani-1]) III; itIs37[pie-1p::mCherry::H2B::pie-1 3'UTR + unc-119(+)] IV*
