# Supplementary material for: C. elegans CLASP/CLS-2 negatively regulates membrane ingression throughout the oocyte cortex and is required for polar body extrusion
Source: PLoS Genet. 2020 Oct 7;16(10):e1008751. doi: 10.1371/journal.pgen.1008751 (PMC7571700; doi:10.1371/journal.pgen.1008751)
Supplement: Attachment — Submitted filename: PLOSGenetics_reviews_w-responses_V2.docx [file pgen.1008751.s047.docx]

28 July 2020

Dear PLOS Genetics Editors,

We are very pleased to re-submit to *PLOS Genetics* our extensively revised Research Article entitled '*C. elegans* CLASP/CLS-2 negatively regulates membrane ingression throughout the oocyte cortex and is required for polar body extrusion'. We also want to again thank you for extending the deadline on these changes, as the extra time has proven very helpful for making extensive revisions and improvements.

As requested, we (1) provide quantitation for all points throughout the text (the places in the text where we needed to specify the number of oocytes analyzed or the scale of the images, and most importantly the quantifications of AIR-2, CYK-4, NMY-2 and ANI-1 levels, with data included in multiple new figure panels); (2) extensively revised the text throughout (all changes are indicated by bold font). These changes include having made what was Figure 3 now Figure 7, due to new data obtained in response to one point raised by the reviewers. We also now state early on in the manuscript (abstract and intro) hypotheses as to the mechanisms by which CLS-2 might influence oocyte meiotic contractile ring assembly. We think this excellent suggestion from Reviewer #2 has led us to improve substantially the flow and thematic structure of the manuscript substantially; (3) we have labeled the supplementary figures as requested.

We also include below a complete set of responses to each point made by all three reviewers, and descriptions of all changes in these responses. Again, the extensive changes to the manuscript text are highlighted throughout in bold font to make them apparent. We have uploaded a Striking image and hope you think it is striking.

We hope you agree that our revised manuscript is much improved. Please let us know if you have any other concerns or needs or questions, and thanks for your help in our effort to publish this work in *PLOS Genetics*!

Sincerely,

Bruce Bowerman, University of Oregon

**Point by point response to all reviewer comments, and description of all changes.**

**Reviewer #1:** In the present manuscript, Schlientz & Bowerman investigate the relationship between spindle assembly and polar body extrusion in C. elegans oocytes. Using novel alleles of the CLASP orthologue CLS-2, the authors show that CLS-2 and katanin (MEI-1) play different roles during polar body extrusion. I particularly liked the idea that late meiosis I events might not be completely linked to early ones. As the authors rightly point out, this is a hard topic to tackle because of early phenotypes obscuring the interpretation of the late ones. However, with a good combination of an accurate description of the results together with the combination of in utero and ex utero experiments, the authors provide very interesting insight into the different roles of key proteins during meiosis, specially CLS-2. Importantly, the experiments are performed using live oocytes, allowing for a proper dynamic assessment of spindle assembly, segregation, and polar body extrusion.

I was very impressed by the level of detail and transparency with which the phenotypes are described and the work the authors took to include all these in the supplementary material.

Overall, I think this work, together with the other recent preprint from the lab, provides very interesting insights into the dynamics of spindle assembly and polar body extrusion in C. elegans oocytes. I would therefore favour publication in PLOS Genetics after some revisions, listed below.
We appreciate these supportive comments and have made substantial improvements to our manuscript based on the helpful suggestions from all three reviewers.

1) In Figure 2E, why does the tubulin quantif. only go back to -340 secs while the images shown go back to -840 sec?

Both the tubulin quantification and the images shown in Figure 2E are from *ex utero* imaging, with live oocytes that were dissected from cut open worms. This was done to maximize the resolution and minimize differences in oocyte position, that occur when oocytes are imaged *in utero*. While we can image oocyte meiosis I from the earliest stages *in utero*, it is much more difficult to isolate very early stage *ex utero* mutant oocytes. The quantification of MT intensity goes back only to -340 seconds because prior that fewer than 9 oocytes were isolated for the earlier time points. We do have imaging data for a small number of oocytes that go back substantially further, and we show still images from the one oocyte that goes back to 840 seconds. The quantified time period (0 to -340 seconds) spans from roughly just prior to anaphase chromosome segregation until the end of meiosis I and beginning of meiosis II in control oocytes. We have modified the text that refers to Figure 2 (such that it no longer indicates quantification throughout meiosis I, but only “during” meiosis I and “during anaphase”), and we also modified the Figure 2 legend to indicate that -340s corresponds to roughly the beginning of anaphase in control oocytes, to clarify the times involved.

2) Figure 3 needs some quantification. Specially in the case of CYK-4, the wild type and mutant look very different and the text only makes a general remark about this.

We thank the reviewer for this suggestion, as it has led us to make substantial changes in the manuscript. We have now quantified the AIR-2 and CYK-4 fluorescence intensity and these data are now included in a new version of Figure 3 (now Figure 7). We also have modified the Results and Discussion extensively to describe these results, as they do raise the possibility that differences in their localization or levels might account at least in part of the observed defects in polar body extrusion in *cls-2* mutants.

3) Specifically, in figure 3A it appears that CLS-2 depletion leads to a phenotype in which there seems to be some degree of chromosome segregation, but then polar body fails. While I am not sure how reproducible this phenomenon is, it would make a good case for stating that polar body extrusion can't happen even when anaphase is not completely affected.

We thank the reviewer for this astute observation about the separation of chromosomes in one of the GFP::AIR-2 expressing oocytes. In a few *cls-2(-)* oocytes, the meiotic cleavage furrow appears to push apart the chromosomal mass (S4 Fig shows two examples of this, in rows 4 and 6). We have never observed any separation of chromosomes that was not associated with such a furrowing event. To avoid confusion, we have replaced the images in Figure 3A with an oocyte that shows the more typical lack of chromosome separation. We also have revised the Results text that refers to S4 Figure and the legend to S4 Figure to point out how we only see such chromosome separation in association with a meiotic cleavage furrow that appears to push the chromosomes apart late in meiosis I. We failed to point this out in our original submission, and we thank the reviewer for prompting to us to more clearly and accurately describe some of our results with these changes.

4) In general, the use of this new cls-2 allele is a nice addition that will also prove useful for other studies. As such, it would be good to have a comparison with RNAi and/or auxin-inducible degradation, because the allele used could have the downside of chronic CLS-2 depletion (i.e. early meiosis defects?).

While we have not ourselves compared our allele phenotypes to those observed after RNAi knockdown, our results are consistent with those reported by other labs that have used RNAi or AID to reduce CLS-2 function. We have improved our citation of these references in the Results section to more fully and explicitly note that our results are consistent with those found using alternative methods to reduce CLS-2 function.

5) One slightly confusing issue is how ‘time = 0’ is defined in the cls-2 mutant. While I acknowledge this will never be an easy task (we routinely have the same issue), the reader would benefit from some clarification to know that the time scales in some experiments are only good for internal reference of each movie and not to be used for comparison between movies.

We agree it is challenging to establish consistent landmarks for the timing of events during *C. elegans* oocyte meiotic cell divisions. As we state in the legend of Figure 2, time = 0 throughout the paper is defined as the end of meiosis I and beginning of meiosis II, when chromosomes left in the oocyte cytoplasm visibly move apart from each other, with exceptions described in figure 4, and in supplemental figures S2-6. For Fig 4 and supplemental figures S2-6, we point out that time = 0 is the point at which membrane furrowing became apparent.

6) We felt that the data on AIR-2 and CYK-4 really needs some quantitation. Specially in the case of CYK-4, the wild type and mutant look very different and the text only makes a general observation from this.

As noted above in response to comment #2, the levels of AIR-2 and CYK-4 have now been quantified, with this new data added to the figures and the text modified substantially to discuss the results.

7) Is the klp-18(RNAi) phenotype 100% penetrant (i.e. are all spindles monopolar)? If so, then disregard. If it isn’t, it would make sense to explicit that the 17/20 cases with PB extrusion come from monopolar spindles and it is not a consequence of partial depletion.

We thank the reviewer for commenting on our lack of clarity with respect to the efficacy of our RNAi knockdowns. As we stated in the Materials and Methods, our *klp-18(RNAi)* was done such that all spindles were monopolar. We have now revised the Materials and Methods and the Results sections to more explicitly explain how we judged this monopolarity by assessing chromosome segregation, which was completely absent after KLP-18 knockdown during both meiosis I and II for our experiments. A spindle pole marker was not used to verify this, but in all cases we never observed any chromosome segregation. While the 17/20 cases of PB extrusion could be a consequence of partial depletion, it still occurs in the absence of normal spindle assembly and a fully penetrant failure in chromosome segregation.

8) ‘Based on the frequent success of polar body extrusion in klp-18 mutants, we conclude that spindle bipolarity and chromosome segregation are not required for polar body extrusion.' This could be a bit of a stretch, since there is a form of segregation-like chromosome movement that could, in theory, provide with whatever mechanism is necessary for PB extrusion. Here again, as mentioned in 1), If you get to see a phenotype with attempt segregation followed by failed PB extrusion, that would be very useful, as it would prove your case.

As noted in response to comment #3, we do not believe any true chromosome segregation occurs in *cls-2* oocytes (only some late separation of chromosomes by an ingressing furrow). And as noted in response to comment #7, the *klp-18(RNAi)* was done such that all oocytes completely failed to segregate chromosomes during meiosis I and II. Nevertheless, as described in our text, we observed a high rate of polar body extrusion after KLP-18 knockdown, despite the fully penetrant loss of chromosome segregation. Similarly, previous work (Connolly et al 2014) has shown that a temperature sensitive allele of *klp-18* can apparently extrude all chromosomes into a polar body, as determined by the absence of a maternal pronucleus in the one-cell stage embryo.

9) For the NMY-2 and ANI-1 movies (which I have to admit I am no expert), it would be helpful to have a measure of chromosome segregation (as a reference only) together with time reference.

As we point out in the text, we assessed NMY-2 and ANI-1 dynamics during the time interval in which global furrowing occurs in both control and *cls-2* mutant oocytes, from roughly the beginning of anaphase to near the end of meiosis I. While a more detailed comparison of chromosome and NMY-2/ANI-1 dynamics might be informative, we have not provided such a comparison, given that we did not detect any obvious changes in NMY-2/ANI-1 dynamics.

10) How do the other steps look in the cls-2 mutants that do extrude a PB? Spindle assembly, chromosome alignment, segregation, etc...? This relates to question 3). Could the authors compare spindle assembly defects in the 15/95 cls-2 mutants movies that do form a polar body vs the 80/95 that don't, using a Fisher test maybe?

This is an interesting question. However, so few *cls-2* mutant oocytes do extrude a polar body that we would have to collect many more additional movies using GFP fusions to make any statistically significant statements as to possible correlations. While we agree this would be interesting, we feel that assessing this is beyond the scope of our current study.

**Reviewer #2:** This manuscript is meant to address the mechanism of polar body formation during C. elegans meiosis. This is a significant problem because polar body formation is conserved in most animal species and because polar body failure results in polyploidy and death. Specifically this manuscript provides high quality phenotypic analysis of polar body formation by time-lapse imaging in a new CRISPR knockout of the microtubule-binding protein CLS-2 and in RNAi depletions of two other microtubule regulators, KLP-18 and MEI-1. Although the results are of high quality, most of the results have either been published previously [polar body failure in cls-2(RNAi) complemented with various RNAi-resistant point mutations (Laband 2017)] or are negative [no change in centralspindlin or acto-myosin in the cls-2 knockout]. In addition, there is no mechanistic hypothesis stated and there is no clear mechanistic conclusion drawn from the results. The use of "we explore" in the final sentence of the abstract suggests that no specific question was asked and that no definitive conclusion was reached in the paper. These issues could potentially be addressed by extensive rewriting of the manuscript. This would require stating a specific question or hypothesis and explaining how the results answer this question and how previously published results could not answer this question.

These comments have indeed led us to extensively rewrite our manuscript. The word “explore” in the abstract has been changed to “discuss.” More importantly, the entire manuscript has been extensively revised, with edits to the abstract, introduction and discussion. We now emphasize throughout a focus on two mechanistic possibilities: (i) CLS-2 regulation of global cortical stiffness is important for oocyte contractile ring assembly and ingression, and (ii) altered relative levels of central spindle proteins in *cls-2* mutant oocytes might disrupt contractile ring assembly and ingression. We believe that these extensive changes, including a nearly complete revision of the Discussion, have greatly improved our manuscript, and we thank the reviewer for motivating us to do so.

Detailed comments below are meant to help clarify the significance of the work to someone reading the manuscript.

We thank the reviewer for the extensive comments that have led us to make substantial improvements that we agree will help clarify the significance of the work.

Line 65: "During anaphase the contractile ring ingresses past both the membrane-proximal pole..." The only reference for this statement is Fig. 1 which is a cartoon with no reference to data in the legend. This statement is also made before the text has narrowed down to C. elegans as a species. In many organisms without an eggshell, the spindle pushes out through the contractile ring. In C. elegans in hyperosmotic conditions where the plasma membrane is pulled away from the eggshell, the spindle also pushes out (Dorn et al., 2011). In utero, the plasma membrane starts pressed against the eggshell and the contractile ring ingresses inward (Fabritius et al., 2011) as stated here. This statement needs to be clarified for both accuracy and proper citation.
We have revised the text in the Introduction to (i) acknowledge the variability of furrow ingression dynamics across species; (ii) specifically refer in *C. elegans* to the dynamics that are observed *in utero*; and (iii) include citations to both Dorn et al 2011 and Fabritius et al 2011.

Line 150: The nature of the CLS-2::GFP should be stated (CRISPRd?, complementing RNAi?) either here or referenced to someplace that it is stated.

We have revised the text in the Results to state the the CLS-2::GFP fusion is an extragenic fusion and cite the source (Maton et al NCB 2015).

Line 154, Fig. 2A: It is stated that metaphase-specific patches of CLS-2::GFP on the entire cortex might suggest that CLS-2 acts on the entire cortex. If this suggestion is going to be stated, it should be clarified whether the patches correspond to linear elements, myosin patches or perhaps clusters of cortical microtubules. If it is not possible to figure this out because of COVID-19, then removing the suggestion that this localization is indicative of global cortical activity might be appropriate.
We have revised the text in the result to state that the patches we observe are likely the same as what have previously been described as rod-shaped structures and linear elements, with appropriate citations.

Line 160: There should be a citation at the end of the first sentence.

Citations have been added

Line 166: "fully penetrant maternal-effect embryonic lethality" would be clearer.

We agree that would be better to assess whether the embryonic lethality is maternal effect. However, homozygous *cls-2* hermaphrodite adults typically rupture and die early in adulthood, expelling their gonad, and thus precluding our ability to purge the worms for mating with wild-type males. We could introduce feminizing mutations to assess this issue but do not consider it to be essential for publication.

Line 194: What is new with the microtubule and chromosome phenotype? The results with the newly generated null allele appear to confirm the previously published RNAi phenotypes which were complemented with RNAi-resistant transgenes in Laband 2017. The new results are thus a second control showing that the phenotypes are not due to off target RNAi effects. The only thing these results show are that Dumont 2010 and Laband 2017 were likely looking at complete rather than partial RNAi depletion. I actually think it is important to reproduce the RNAi work of others using a CRISPR knockout but this needs to be clearly explained to the reader.

As noted in responses to Reviewer #1, we have clarified that the results with our CRISPR alleles are consistent with previous studies using RNAi and AID knockdowns. Nevertheless, our data from likely null alleles provides an important confirmation of results previously described using less conclusive approaches. Furthermore, our analysis of the pole marker GFP::ASPM-1 significantly advances our understanding of the spindle assembly defect in *cls-2* mutants. While we view our analysis of the *cls-2(-)* spindle defects as being more comprehensive compared to previous work, our focus has been on characterizing in detail the *cls-2(-)* polar body extrusion defects. Our initial analysis of the meiotic spindle defects confirm previous studies, provides some advances, and sets the stage for our extensive analysis of polar body extrusion.

Line 201: What is new about the polar body phenotype? It seems like less of an advance than the Laband 2017 results which included polar body phenotypes with point mutations in specific TOG domains.

While Dumont et al 2010, Laband et al 2017, and Pelisch et al 2019 have all previously noted the absence of polar body extrusion in oocytes depleted of *cls-2*, they did not describe the polar body extrusion defects in any detail beyond the rate of failure and the requirement for specific TOG-domain residues. We provide an extensive image analysis of membrane ingression using the PH domain marker, and also by imaging the dynamics of non-muscle myosin NMY-2 and the anillin ANI-1. Moreover, we provide an extensive comparison to other spindle-defective mutants to show that CLS-2 has unique and important roles in regulating the assembly and function of the contractile ring during polar body extrusion. We believe these are important advances that are rigorously documented and provide novel insights into the poorly understood process of polar body extrusion during oocyte meiotic cell division.

The description of the assay for polar body extrusion failure is in the Methods and makes it unclear whether there is a defect in extrusion of the second polar body. If this information can be gleaned from existing data, this would at least be a slight advancement over previously reported polar body failure phenotypes. Line 240 speculates that mei-1 mutants might have a meiosis II polar body defect which suggests that the time-lapse imaging in this study did not go through completion of meiosis II. This manuscript thus does not resolve the previously published large polar body phenotype with the current no polar body phenotype for mei-1. A standard in this field would be to report polar body number and size in mitotic embryos. This would be especially important for klp-18(RNAi) which is reported to succeed in polar body formation. An-1 knockdown polar bodies do not reveal their failure until very late. This manuscript also uses RNAi by feeding for mei-1 whereas previous studies have used allelic series of mei-1.

We have revised the Methods (Image Analysis, Quantification, and Statistical Analysis section) to explicitly state that we only assessed polar body extrusion during meiosis I. We agree that it would be interesting to examine meiosis II polar body extrusion, although any such defects could be indirectly caused by the earlier defects during meiosis I, and such an analysis is beyond the scope of this manuscript. As noted in the previous comment, we believe that our detailed characterization of polar body extrusion during meiosis I in three different mutants, and in control oocytes, goes well beyond any previous characterizations of polar body extrusion for these mutants and constitutes an important contribution.

The AIR-2 and myosin dynamics results are all negative. Some negative results would be fine if a positive result was reported that yielded some clue as to why cls-2 knockouts do not make polar bodies.

As noted in response to the comments from Reviewer #1, we have now quantified the levels of AIR-2 and CYK-4 fluorescence and include these in revised figures. We fully agree this was important to do, and the results do show a difference in the *cls-2* mutants that may explain why polar body extrusion fails. We have extensively revised our manuscript to take into account these new data. We moved the section in the Results describing our analysis of AIR-2 and CYK-4 to the end of the Results, and we now provide new text in the Discussion as to the potential significance of these findings.

Line 244: The conclusion that spindle bipolarity and chromosome segregation are not required for polar body extrusion because 15/20 klp-18(RNAi) embryos successfully extruded a polar body (Fig. 2G) could be a significant result but is only weakly supported. The successful polar body extrusion could be due to partial RNAi. Filming with transgenes (GFP::ASPM-1) that convincingly showed a lack of spindle bipolarity during late anaphase would be required to address this issue. Mullen and Wignall (2017) presented evidence that klp-15/16-depleted embryos have no bipolarity at metaphase but assemble a midzone during anaphase. Filming GFP:SPD-1 in klp-18(RNAi) might resolve whether this is happening during klp-18(RNAi) polar body formation. I am not suggesting that the authors do experiments during quarantine. I am just pointing out the brevity of the results supporting this conclusion in the manuscript. The novelty/lack of novelty of monopolar spindle polar body formation could be cited more extensively to Canman et al. (2003) and others have shown that monopolar spindles can induce mitotic cleavage furrows but there is no discussion of this.

As noted above in responses to comments #7 and #8 from Reviewer 1, we have revised the text in the Methods to more explicitly point out that our knockdowns of *klp-18* and *mei-1* result in fully penetrant defects in chromosome segregation, to more clearly explain our conclusion that the polar body extrusion defects observed in *cls-2* mutants are not due simply to the failure in chromosome segregation. We agree this is an important finding. Also, we now refer to the relevant work from Canman et al 2003 in the Discussion.

Regarding the conclusion that CLS-2 directly suppresses global cortical furrowing. CLS-2 depletion reduces microtubule density in the spindle (this study) and CLS-2 depletion blocks microtubule-dependent cytoplasmic streaming (Yang et al., 2003) and microtubule-dependent yolk granule packing (McNally et al., 2010) throughout the embryo. There should be a clearer discussion of the possibility that reduced density of cortical microtubules causes the global increase in furrowing. Microtubule-dependent suppression of cortical furrowing has been reported many times including tubulin(RNAi) in C. elegans meiosis (Fabritius et al., 2011) and during mitosis and could be more clearly discussed. This is an area that could be developed into a hypothesis that is stated at the beginning of the paper.

We thank the reviewer for these interesting and helpful suggestions. In our revised Discussion, we now refer to the possible involvement of cytoplasmic streaming and yolk granule packing in oocytes depleted of CLS-2. We also have revised the Abstract, the last paragraph of the Introduction, and the opening and other parts of the Discussion to explicitly state hypotheses as to how CLS-2 influence contractile ring assembly and ingression during *C. elegans* oocyte meiotic cell division. In our original submission, we avoided emphasizing mechanistic ideas early on, given our lack of conclusive insight in the molecular mechanism through which CLS-2 influences polar body extrusion. But we agree that our manuscript has been improved by adding text throughout to provide a mechanistic context for thinking about our findings.

**Reviewer #3:** Schlientz and Bowerman addressed the relationship between meiotic spindle assembly and polar body extrusion. They characterized three spindle assembly mutants, cls-2, klp-18, and mei-1. Mutants of CLS-2 fail to form bipolar spindles, had low levels of microtubules, and failed to segregate DNA. Previous work showed that mutants of KLP-18 form monopolar spindles and fail to segregate chromosomes. Mutants of MEI-1 form apolar spindles and fail to segregate chromosomes. Mutants of CLS-2 mostly do not extrude polar bodies. Live imaging shows that there are defects in ingression and they have abnormal assembly of the NMY2:::GFP contractile rings. The mei-1 mutants mostly do not form polar bodies, but the defect is later than in cls-2 mutants. The oocytes initially ingress but fail later. Many of the contractile rings assemble and initiate constriction but then fail. The klp-18 mutants mostly do form polar bodies, suggesting that bipolar spindle formation and chromosome segregation is not required for polar body formation. In the absence of CLS-2, there were more global cortical furrows, suggesting that CLS-2 negatively regulates furrowing.

This manuscript is mainly descriptive, and at times this can be a bit frustrating. For example, there are many defects in the cls-2 mutants and it is difficult to discern whether the later defects in contractile ring assembly are due to earlier defects in ingression. Or, even in understanding how these regulators of spindle assembly affect these processes and why the phenotypes are so variable.

We indeed have ourselves struggled with the variability of the defects in the different mutant oocytes (not just in *cls-2* mutants!). This variability ultimately led us to include supplemental figures that show the defects in each examined oocyte. These supplemental figures clearly document the variability, and we have done our best to minimize the categories when describing and grouping the observed phenotypes. Given our use of CRISPR-generated *cls-2* null mutants, and the variability observed even in control oocytes, we believe that this phenotypic variability is more likely a reflection of the nature of the process, and the challenges of imaging it, and not due to variable reduction in gene function.

But, overall the live cell analysis of the steps of polar body formation provide a nice and important understanding of how/when the processes fail in the different mutants. And, this study provides a foundation for future more mechanistic studies. The major findings of this study include: i) spindle bipolarity and chromosome segregation are not required for polar body contractile ring formation and chromosome extrusion; ii) CLS-2 is required for early contractile ring assembly; iii) MEI-1 is needed for full contractile ring constriction; and, iv) CLS-2 negatively regulates membrane ingression.

We thank the reviewer for these supportive comments and agree that these are important findings.

Overall, I have only minor suggestions for improvements to the manuscript:

1) The authors propose that CLS-2 prevent membrane ingressions at the oocyte cortex. Do the CLS-2::GFP patches co-localize with actin filaments?

We do not know if the CLS-2::GFP patches co-localize with actin, or with patches of NMY-2::GFP. Unfortunately, we were unable to obtain reproducible high quality imaging with an array of live actin markers during oocyte meiotic cell division. We agree these are important issues and point out in the Discussion that they are important areas for future investigation.

2) Can the authors provide the data (in graph form) showing the dynamics of NMY-2::GFP and mNG::ANI-1 in control and mutant oocytes?

We thank the reviewer for suggesting this, and we have added an additional supplementary figure (S19 Fig) with graphs showing the percentage of oocyte cortex occupied by patches of NMY-2 and ANI-1 over time. The results support our tentative conclusions in the original submission that there are not obvious changes in the dynamics of NMY-2 and ANI-1 during polar body extrusion.

3) It seems interesting that the mutants can MEI-1 mutants can initiate polar body formation and then regress back to no polar body formation. Can the authors further speculate on the regulation of how this occurs?

In the Discussion, we now speculate that the defects observed in *mei-1* mutants may be related to changes in the localization or function of the centralspindlin complex (MgcRacGAP/CYK-4 and kinesin-6/ZEN-4), as the mei-1 phenotype is reminiscent of what has been reported for oocytes depleted of either cyk-4 or zen-4 (Fabritius et al 2011). We also now note that the late defect could be due to a requirement for microtubule severing, similar to what has been reported for *mei-1* severing defective mutants during meiosis II (Gomes et al 2013).

4) For clarity, it would greatly help the reader if the figures were labeled with the mutants that are being described so that the reader does not need to refer back to the figure legends. This has been done in the main figures, but not in most of the supplementary figures (Figures S3- S12, S14-18).

We have added labels to these figures.

5) In Figures 7A and S12-S13, it would be helpful to point out where the spindle was localized.

Arrowheads have been added to figure 7A and S11-13 to indicate approximate spindle/spindle associated furrow position

6) Correct typo on line 533.

Typo has been corrected

**Have all data underlying the figures and results presented in the manuscript been provided?**
Large-scale datasets should be made available via a public repository as described in the *PLOS Genetics* [data availability policy](https://urldefense.com/v3/__http:/journals.plos.org/plosgenetics/s/data-availability__;!!C5qS4YX3!RpPwlEg0Cr3oH2kOMld2-HXqlf9WoWcp8vK0Cu04W5mTQSzY64SOm0Z9wj9pagp41GQ$), and numerical data that underlies graphs or summary statistics should be provided in spreadsheet form as supporting information.

Reviewer #1: Yes

Reviewer #2: Yes

Reviewer #3: Yes

PLOS authors have the option to publish the peer review history of their article ([what does this mean?](https://urldefense.com/v3/__https:/journals.plos.org/plosgenetics/s/editorial-and-peer-review-process*loc-peer-review-history__;Iw!!C5qS4YX3!RpPwlEg0Cr3oH2kOMld2-HXqlf9WoWcp8vK0Cu04W5mTQSzY64SOm0Z9wj9pAvjFRTg$)). If published, this will include your full peer review and any attached files.


If you choose "no", your identity will remain anonymous but your review may still be made public.


**Do you want your identity to be public for this peer review?** For information about this choice, including consent withdrawal, please see our [Privacy Policy](https://urldefense.com/v3/__https:/www.plos.org/privacy-policy__;!!C5qS4YX3!RpPwlEg0Cr3oH2kOMld2-HXqlf9WoWcp8vK0Cu04W5mTQSzY64SOm0Z9wj9ploXhCec$).

Reviewer #1: Yes: Federico Pelisch

Reviewer #2: No

Reviewer #3: No
